# Analysis of Gene Expression Profiles of Soft Tissue Sarcoma Using a Combination of Knowledge-Based Filtering with Integration of Multiple Statistics

**DOI:** 10.1371/journal.pone.0106801

**Published:** 2014-09-04

**Authors:** Anna Takahashi, Robert Nakayama, Nanako Ishibashi, Ayano Doi, Risa Ichinohe, Yoriko Ikuyo, Teruyoshi Takahashi, Shigetaka Marui, Koji Yasuhara, Tetsuro Nakamura, Shintaro Sugita, Hiromi Sakamoto, Teruhiko Yoshida, Tadashi Hasegawa, Hiro Takahashi

**Affiliations:** 1 Plant Biology Research Center, Chubu University, Kasugai, Aichi, Japan; 2 Division of Genetics, National Cancer Center Research Institute, Tokyo, Japan; 3 Department of Orthopaedic Surgery, Keio University School of Medicine, Tokyo, Japan; 4 Division of Biological Science, Graduate School of Science, Nagoya University, Nagoya, Aichi, Japan; 5 Faculty of Horticulture, Chiba University, Matsudo, Chiba, Japan; 6 Graduate School of Horticulture, Chiba University, Matsudo, Chiba, Japan; 7 Graduate School of Bioscience and Biotechnology, Chubu University, Kasugai, Aichi, Japan; 8 Department of Surgical Pathology, Sapporo Medical University School of Medicine, Sapporo, Hokkaido, Japan; 9 Pathology Division, National Cancer Center Hospital, Tokyo, Japan; University of Louisville, United States of America

## Abstract

The diagnosis and treatment of soft tissue sarcomas (STS) have been difficult. Of the diverse histological subtypes, undifferentiated pleomorphic sarcoma (UPS) is particularly difficult to diagnose accurately, and its classification per se is still controversial. Recent advances in genomic technologies provide an excellent way to address such problems. However, it is often difficult, if not impossible, to identify definitive disease-associated genes using genome-wide analysis alone, primarily because of multiple testing problems. In the present study, we analyzed microarray data from 88 STS patients using a combination method that used knowledge-based filtering and a simulation based on the integration of multiple statistics to reduce multiple testing problems. We identified 25 genes, including hypoxia-related genes (e.g., *MIF*, *SCD1*, *P4HA1*, *ENO1*, and *STAT1*) and cell cycle- and DNA repair-related genes (e.g., *TACC3*, *PRDX1*, *PRKDC*, and *H2AFY*). These genes showed significant differential expression among histological subtypes, including UPS, and showed associations with overall survival. *STAT1* showed a strong association with overall survival in UPS patients (logrank *p* = 1.84×10^−6^ and adjusted *p* value 2.99×10^−3^ after the permutation test). According to the literature, the 25 genes selected are useful not only as markers of differential diagnosis but also as prognostic/predictive markers and/or therapeutic targets for STS. Our combination method can identify genes that are potential prognostic/predictive factors and/or therapeutic targets in STS and possibly in other cancers. These disease-associated genes deserve further preclinical and clinical validation.

## Introduction

Recent advances in genomic technologies offer an excellent opportunity to determine the complete biological characteristics of neoplastic tissues, resulting in improved diagnosis, treatment selection, rational classification based on molecular carcinogenesis, and identification of therapeutic targets. The diagnosis and treatment of soft tissue sarcomas (STS) have been difficult because STSs comprise a group of highly heterogeneous tumors in terms of histopathology, molecular signature, histological grade, and primary site. These tumors have generally been classified into subtypes according to their histological resemblance to normal tissue. The Fédération Francaise des Centres de Lutte Contre le Cancer (FNCLCC) grading system was defined more than 20 years ago and is still the most commonly used grading system for STS [1], [2]. Treatment of STS is based on both histological subtype and histological grade. The understanding gained regarding the molecular pathology of cancer in recent decades suggests that some tumor types exhibit stand-alone recurrent genetic aberrations, such as chromosomal translocations, that result in gene fusions, e.g., *SYT*-*SSX* in synovial sarcoma (SS) [3], *TLS*-*CHOP* in myxoid/round cell liposarcoma (MLS) [4], and *KIF5B*-*RET* in lung adenocarcinoma [5], or somatic mutations, e.g., *KIT* in gastrointestinal stromal tumors (GIST) [6] and 26 mutated genes (*TP53*, *KRAS*, *EGFR*, and 23 other genes) in lung adenocarcinoma [7]. The molecular markers specific to each tumor type are useful for tumor classification [8]. In contrast, several malignant tumors, such as malignant fibrous histiocytoma (MFH), are characterized by numerous nonrecurrent, complex chromosomal aberrations, and they frequently show overlapping histological features and immunophenotypes that are difficult for pathologists to interpret [9]. In particular, the diagnosis of MFH has been a controversial issue [10]–[13]. MFH is the most common soft tissue sarcoma in adults. It has a wide range of histological subtypes [13]. For this reason, discrimination between MFH and other STSs is difficult, but this discrimination is necessary because there are significant differences in the 5-year survival rates of the STS subtypes [14]: 100% for well-differentiated liposarcoma (WLS), 71% for synovial sarcoma (SS), 46% for pleomorphic MFH, and 92% for myxofibrosarcoma (MFS). MFH was renamed undifferentiated pleomorphic sarcoma (UPS) in 2002 by the World Health Organization (WHO) [15]. MFS was considered a subtype of MFH before this classification; WHO reclassified MFS as another subtype of STS [15]. Discrimination between UPS and MFS is particularly difficult [14] because of their histological similarities and because of the considerable heterogeneity of UPS [13]. UPS was previously characterized by global gene expression analysis using analysis of variance (ANOVA) and clustering analysis [13]. Although some possible prognostic factors were identified, the list of factors was not complete because the study was conducted without information on patient outcomes. In the present study, we hypothesized that some genes can serve both as diagnostic markers for histological subtyping and as prognostic markers of overall survival in STS. We used a combination of statistical and bioinformatic methods to identify those genes.

Many statistical and bioinformatic methods have been proposed for global biological information analysis in the past 3 decades. For example, basic local alignment search tool (BLAST) [16], ClustalW [17], BLAST-based algorithm for the identification of upstream ORFs with conserved amino acid sequences (BAIUCAS) [18], and G4 DNA motif region finder by R (G4MR-FindeR) [19] have been used for sequence analysis; hierarchical clustering [20], fuzzy k-means [21], and fuzzy adaptive resonance theory (FuzzyART) [22], [23] have been used for gene cluster analysis; gene set enrichment analysis (GSEA) [24], modified signal-to-noise (S2N′) [25], and projective adaptive resonance theory (PART) [26], [27] have been used for gene selection; fuzzy neural network (FNN) [28], [29] and boosted fuzzy classifier with a SWEEP operator (BFCS) [30]–[32] have been used for the construction of prediction models; and IntPath [33] and Stringent DDI-based Prediction [34] were used for analysis of pathways and protein–protein interactions. The use of statistical or bioinformatic analysis is practical and useful for clinical diagnosis [35]–[37] and the identification of marker genes [38]–[43]. In the present study, we focused on microarray data analysis; however, the analysis of data obtained using next-generation sequencing technologies [44] is a subject of an upcoming project.

Global analysis of gene expression is a powerful method for the identification of prognostic/predictive factors and/or therapeutic targets. However, it is often difficult, if not impossible, to identify definitive disease-associated genes using genome-wide analysis alone, primarily because of multiple testing problems. In this situation, knowledge-based approaches, such as knowledge-based fuzzy adaptive resonance theory (KB-FuzzyART) [45] and knowledge-based single nucleotide polymorphism (KB-SNP) [46], [47], are effective and interpretable [48]–[50]. Online Mendelian Inheritance in Man (OMIM) is a continuously updated catalog of human genes and genetic disorders and traits. In the present study, we used OMIM as a knowledge source for narrowing the list of candidate genes and applied the OMIM-based method to gene expression data from STS patients. Thus, we identified 25 genes that showed significant differential expression among histological subtypes, including UPS, and showed associations with overall survival. According to the literature, these genes are useful not only as diagnostic markers for the discrimination of molecular pathway-based subtypes but also as prognostic/predictive markers and/or therapeutic targets for STS. Moreover, these genes are useful for understanding the mechanisms underlying tumor progression or metastasis and for the rational design of anticancer therapeutics. Therefore, our combination method of knowledge-based filtering and simulation based on the integration of multiple statistics can identify potential prognostic/predictive factors and/or therapeutic targets in STS and possibly in other cancers.

## Materials and Methods

### Ethics statement

The study was conducted according to the principles expressed in the Declaration of Helsinki. The ethics committee of the National Cancer Center approved the study protocol. All patients provided written informed consent.

### Patients and tumor samples

The characteristics of the 88 STS patients (20 with UPS, 15 with MFS, 17 with SS, 20 with myxoid liposarcoma [MLS], 6 with leiomyosarcoma [LMS], 5 with fibrosarcoma [FS], and 5 with a malignant peripheral nerve sheath tumor [MPNST]) enrolled in this study are shown in Table 1. All patients had received a histological diagnosis of primary soft tissue tumor at the National Cancer Center Hospital, Tokyo, between 1996 and 2002 [51], as shown in Table S1. Tumor samples were obtained at the time of excision and were cryopreserved in liquid nitrogen.

**Table 1:** Characteristics of the 88 patients with soft tissue sarcoma.

Characteristics		STS patients (*n* = 88)
Gender	Male	46
	Female	42
Age	Median	54
	MAD	19
Histological type	UPS	20
	MLS	20
	SS	17
	MFS	15
	LMS	6
	FS	5
	MPNST	5
Histological grade	1	14
	2	23
	3	51
Relapse events	Metastasis	43

STS: soft tissue sarcoma, MAD: Median absolute deviation, UPS: undifferentiated pleomorphic sarcoma, MLS: myxoid liposarcoma, SS: synovial sarcoma, MFS: myxofibrosarcoma, LMS: leiomyosarcoma, FS: fibrosarcoma, MPNST: malignant peripheral nerve sheath tumor.

### Microarray analysis

For RNA extraction, trained pathologists carefully excised the tissue samples from the main tumor, leaving a margin free from the surrounding nontumorous tissue. The elimination of nontumorous stromal cells is necessary for gene expression analysis of carcinomas because tumor tissues contain a significant number of nontumorous stromal cells, including fibroblasts, endothelial cells, and inflammation-associated cells. STS contains non-tumorous stromal cells that are difficult to exclude because STS originates from mesenchymal cells. However, in STS, the tumor tissue contains very few non-tumorous stromal cells and therefore unlikely to confound the analysis. Hence, laser microdissection was not performed in this study. Total RNA samples extracted from the bulk tissue specimens were labeled with biotin and hybridized to high-density oligonucleotide microarrays (Human Genome U133A 2.0 Array; Affymetrix, Santa Clara, CA, USA) comprising 22,283 probe sets representing 18,400 transcripts, according to the manufacturer’s instructions. The scanned array data were processed using the Affymetrix Microarray Suite v.5.1 software (MAS5), which scaled the average intensity of all the genes on each array to the target signal of 1000. The microarray data from the present study are available in the Genome Medicine Database of Japan (GeMDBJ) [52] (https://gemdbj.nibio.go.jp/dgdb/) under the accession number EXPR058P.

### Data preprocessing

**Figure 1:**
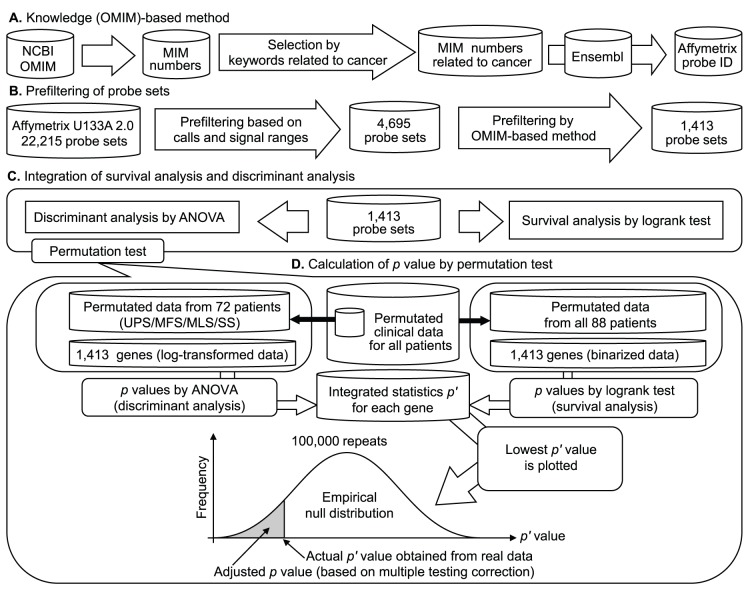
A schematic of gene selection and the simulation based on the permutation test. (A) The knowledge (OMIM)-based method. The list of OMIM numbers related to cancer (e.g., cancer, carcinoma, sarcoma, tumor, and neoplasm) was selected and converted into Affymetrix probe IDs in Ensembl. (B) Prefiltering of probe sets. This procedure was based on the number of absent calls and the range of signals. A signal range (95th percentile to 5th percentile) of >2000 was used as a percentile filter. Furthermore, we excluded probe sets for which the number of absent calls was >50% (44/88). Probe sets related to cancer were selected using the OMIM-based method. (C) Integration of survival analysis and discriminant analysis. (D) Clinical data from all patients were permutated. Permutated data for 72 STS patients (20 UPS, 15 MFS, 20 MLS, and 17 SS patients) were extracted from the permutated data of all patients. For these data, *p* values (*p*_1_) were calculated by applying ANOVA to the log-transformed gene expression data to discriminate among UPS, MFS, MLS, and SS. In addition, permutated data from 88 patients were used for survival analysis. For these data, *p* values (*p*_2_) were calculated by applying the logrank test to the binarized gene expression data to analyze the outcomes in the STS group. The integrated statistic *p*′ was defined as *p*_1_×*p*_2_. The lowest *p*′ value was selected for each repetition. This procedure was repeated 100,000 times, and an empirical null distribution was constructed. Using the distribution, the actual *p*′ value obtained from the real data was converted to the adjusted *p* value (based on the correction for multiple testing problems).

We excluded 68 control probe sets and 2343 genes that were subject to cross-hybridization according to NetAffx Annotation (www.affymetrix.com). Furthermore, we excluded those genes for which more than 50% (44/88) of the samples showed an absent call (i.e., the detection call determined by MAS5 based on the *p* value of the one-sided Wilcoxon signed-rank test; an absent call corresponds to *p*≥0.065, which is the default threshold in MAS5). An absent call indicates that the expression signal was undetectable. Genes showing low variance, i.e., a signal range value (95th percentile to 5th percentile) of less than 2000, were excluded [40]. Furthermore, we conducted an OMIM-based reduction of the number of candidate genes. In total, 1412 genes were selected, to which we applied log-transformation or binarization using the median value as a threshold for each gene, as shown in Fig. 1. The 2 types of datasets, log-transformed and binarized, were used for ANOVA and the logrank test, respectively.

### Simulation based on the combination of a permutation test and the integration of multiple statistics

We previously proposed a statistical simulation based on a permutation test and the integration of multiple statistics [51]. This method was used in the present study. We first calculated *p* values using ANOVA to discriminate among histological subtypes, including UPS, MFS, SS, and MLS. We also calculated *p* values by means of the logrank test in the survival analysis of all STS patients in relation to the 1412 filtered genes. We defined the integrated statistic *p*′ as *p*_1_×*p*_2_, where *p*_1_ is the *p* value from ANOVA and *p*_2_ is the *p* value from the logrank test. The same STS patients (*n* = 72; 20 UPS, 15 MFS, 17 SS, and 20 MLS patients) were used in both of these tests. The integrated statistic *p*′ could be underestimated by the use of 72 common samples. Therefore, to cancel this influence, we conducted a simulation based on the permutation test, as shown in Fig. 1, to estimate the adjusted *p*′ values as well as the multiple testing problems.

### Statistical analysis

The median value of the gene expression signals for each gene was calculated, and the patients were distributed into 2 groups using the median value as a threshold for each gene. Logrank tests [53] were performed for overall survival of STS patients for each gene. We also calculated Spearman’s rank correlation coefficients to assess the relationships between gene expression signals and histological grades [54] or incidence of tumor metastases. We considered data obtained after 50 months of follow-up as censored data in the analysis of the logrank test, similar to the procedure followed in our previous study [51]. Kaplan-Meier curves [55] based on histological subtype were constructed for all STS patients.

### OMIM

OMIM is a continuously updated catalog of human genes and genetic disorders and traits, with a focus on the molecular relationship between genetic variation and phenotypic expression. The list of MIM gene accession numbers associated with keywords related to cancer was obtained from the OMIM website (http://www.omim.org/). We used several keywords related to cancer, including “cancer,” “carcinoma,” “sarcoma,” “tumor,” and “neoplasm,” to create the MIM gene accession number list. There were 4394 MIM gene accession numbers, as shown in Table S2. The final MIM gene accession number list was obtained on January 10, 2014.

### Ensembl

Ensembl is a joint project between EMBL-EBI and the Sanger Centre to develop software that produces and maintains automatic annotation of eukaryotic genomes [56]. We converted MIM numbers to the Affymetrix probe set IDs of the Human Genome U133A 2.0 Array using information retrieved from Ensembl on January 10, 2014. There were 5155 Affymetrix probe set IDs, as shown in Table S3.

### Principal component analysis (PCA)

We used PCA to reduce the gene expression profile data to a two-dimensional dataset. PCA was first proposed in 1901 by Pearson [57]. This method is a statistical procedure that uses orthogonal transformation to convert a set of observations of possibly correlated variables into a set of values of linearly uncorrelated variables called principal components (PCs). The number of PCs is less than or equal to the number of original variables. This transformation is defined in such a way that the first PC has the greatest possible variance.

### Multiple testing correction

The Bonferroni correction is a method used to address the problem of multiple comparisons (also known as the multiple testing problem). It is considered the simplest and most conservative method for control of the family-wise error rate (FWER). False discovery rate (FDR) controlling procedures, such as the Benjamini-Hochberg (BH) method [58], are more powerful (i.e., less conservative) than the FWER procedures, but their use increases the likelihood of false positives within the rejected hypothesis. In the present study, the BH method was used to calculate the *q* value. The *q* value is defined as an FDR analog of the *p* value.

### Heatmap and hierarchical clustering analyses

A heatmap was created using the R program (function heatmap.2 in Package gplots) for the log-transformed and scaled gene expression data of selected genes. Hierarchical clustering was also conducted using the Euclidean distance and complete linkage (default parameters of function heatmap.2).

## Results

### Kaplan-Meier curves for 4 histological subtypes

**Figure 2:**
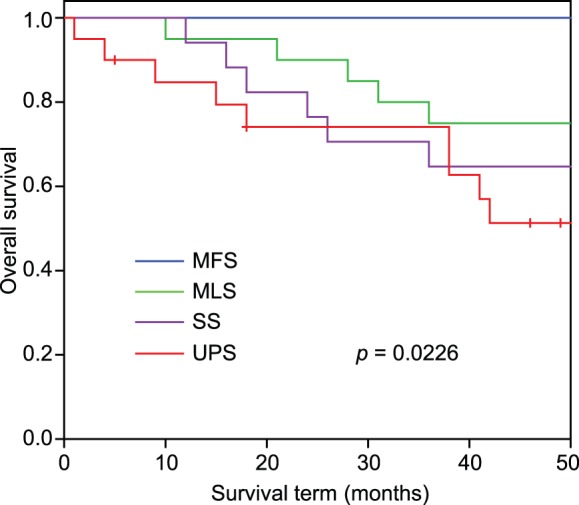
Kaplan-Meier curves for 4 histological types of STS. *P* value was calculated by logrank test. UPS: undifferentiated pleomorphic sarcoma, MLS: myxoid liposarcoma, SS: synovial sarcoma, MFS: myxofibrosarcoma.

Kaplan-Meier curves based on a histological subtype were constructed for all STS patients, as shown in Fig. 2. This figure shows that MFS had a good prognosis, MLS and SS had intermediate prognoses, and UPS had a poor prognosis. Although the logrank test yielded statistically significant results (*p*<0.05) in histological types, we conducted gene expression analysis to select molecular markers for more accurate diagnosis in accordance with the analysis.

### Extraction of genes that are both diagnostic and prognostic markers, by means of a simulation using the permutation test

To extract genes that are both diagnostic markers (for discrimination of histological subtypes) and prognostic markers (of overall survival in STS), we applied a simulation based on the combination of a permutation test and the integration of multiple statistics into 1412 prefiltered probe sets of microarray data obtained from STS patients. As shown in Table 2, 29 probe sets, representing 25 genes, were extracted (adjusted *p* value <0.05).

**Table 2:** Genes extracted using the simulation based on the permutation test.

Affymetrix probe ID	Accession no.	Gene symbol	*p* value		Integrated statistics *p*′	Adjusted *p* value
			ANOVA	Log-rank test		
200832_s_at	AB032261	*SCD1*	2.47E-06	6.06E-03	1.50E-08	6.70E-04
200887_s_at	NM_007315	*STAT1*	1.17E-04	1.91E-02	2.24E-06	3.59E-02
201231_s_at	NM_001428	*ENO1/MBP1*	2.27E-08	1.06E-03	2.40E-11	<1.00E-05
201508_at	NM_001552	*IGFBP4*	3.21E-06	4.01E-02	1.29E-07	3.76E-03
202236_s_at	NM_003051	*SLC16A1/MCT1*	1.12E-04	6.93E-04	7.77E-08	2.34E-03
202870_s_at	NM_001255	*CDC20*	9.26E-07	6.28E-03	5.81E-09	2.90E-04
203065_s_at	NM_001753	*CAV1*	1.33E-10	3.28E-02	4.35E-12	<1.00E-05
203323_at	BF197655	*CAV2*	5.67E-10	2.35E-02	1.33E-11	<1.00E-05
203554_x_at	NM_004219	*PTTG1*	7.33E-09	5.64E-03	4.13E-11	<1.00E-05
207011_s_at	NM_002821	*PTK7*	2.57E-07	1.89E-02	4.86E-09	2.70E-04
207168_s_at	NM_004893	*H2AFY/H2AX*	2.83E-05	1.80E-02	5.11E-07	1.19E-02
207543_s_at	NM_000917	*P4HA1*	1.06E-08	5.73E-04	6.06E-12	<1.00E-05
208680_at	L19184	*PRDX1*	5.73E-08	1.64E-02	9.37E-10	6.00E-05
208694_at	U47077	*PRKDC/DNA-PKcs*	1.71E-04	1.31E-02	2.25E-06	3.60E-02
208767_s_at	AW149681	*LAPTM4B*	5.47E-05	1.65E-02	9.04E-07	1.81E-02
209030_s_at	NM_014333	*CADM1/TSLC1*	1.80E-10	4.20E-02	7.59E-12	<1.00E-05
209031_at	AL519710	*CADM1/TSLC1*	2.10E-11	5.68E-03	1.19E-13	<1.00E-05
209543_s_at	M81104	*CD34*	2.66E-06	1.54E-02	4.10E-08	1.33E-03
210495_x_at	AF130095	*FN1*	3.90E-08	1.78E-02	6.96E-10	2.00E-05
210559_s_at	D88357	*CDK1/CDC2*	7.69E-07	4.30E-02	3.31E-08	1.14E-03
212097_at	AU147399	*CAV1*	1.54E-09	2.95E-03	4.53E-12	<1.00E-05
212464_s_at	X02761	*FN1*	1.93E-08	1.78E-02	3.44E-10	1.00E-05
217294_s_at	U88968	*ENO1/MBP1*	8.81E-08	2.33E-02	2.05E-09	1.50E-04
217871_s_at	NM_002415	*MIF*	5.67E-08	1.46E-02	8.29E-10	5.00E-05
218308_at	NM_006342	*TACC3*	2.82E-05	2.26E-02	6.38E-07	1.40E-02
218502_s_at	NM_014112	*TRPS1*	1.48E-18	3.99E-02	5.90E-20	<1.00E-05
218755_at	NM_005733	*KIF20A/MKlp2*	3.01E-06	2.02E-02	6.08E-08	1.94E-03
219918_s_at	NM_018123	*ASPM*	1.22E-05	1.64E-02	2.00E-07	5.51E-03
220942_x_at	NM_014367	*FAM162A/HGTD-P*	4.44E-05	3.21E-02	1.42E-06	2.56E-02

Adjusted *p* values were calculated using the permutation test (100,000 repeats).

### Association analysis of the histological grade (or metastasis status) and gene expression data for the 25 selected genes

We next used Spearman’s rank correlation analysis to analyze the association between the gene expression level in STS patients and the histological grade (or metastasis status), as shown in Table 3. Table 3 shows that genes with positive *ρ* were upregulated in highly malignant tumors, whereas genes with negative *ρ* were downregulated in highly malignant tumors. The expression levels of almost all of the 25 genes were associated with either the histological grade or metastasis. However, stearoyl-CoA desaturase 1 (*SCD1*) and signal transducer and activator of transcription 1 (*STAT1*) were not associated with either the histological grade (*SCD1*: *ρ* = −0.0191, *p = *0.860; *STAT1*: *ρ* = −0.146, *p = *0.173) or metastasis (*SCD1*: *ρ* = 0.0237, *p = *0.826; *STAT1*: *ρ* = −0.177, *p = *0.0995). This result indicates that *SCD1* and *STAT1* expression levels can be related to the overall survival of STS patients but not to metastasis. Therefore, these data suggest that *SCD1* and *STAT1* expression levels can be used in combination with the histological grade to predict the survival of STS patients.

**Table 3:** Correlation analysis based on Spearman’s rank correlation coefficient between gene expression data and the histological grade (or metastasis status).

Affymetrixprobe ID	Accession no.	Gene symbol	With histological grade		With metastasis	
			*ρ*	*p* value	*ρ*	*p* value
200832_s_at	AB032261	*SCD1*	−0.0191	8.60E-01	0.0237	8.26E-01
200887_s_at	NM_007315	*STAT1*	−0.146	1.73E-01	−0.177	9.95E-02
201231_s_at	NM_001428	*ENO1/MBP1*	0.356	6.66E-04	0.247	2.01E-02
201508_at	NM_001552	*IGFBP4*	−0.247	2.04E-02	−0.211	4.87E-02
202236_s_at	NM_003051	*SLC16A1/MCT1*	0.400	1.12E-04	0.341	1.17E-03
202870_s_at	NM_001255	*CDC20*	0.413	6.27E-05	0.204	5.65E-02
203065_s_at	NM_001753	*CAV1*	−0.250	1.87E-02	−0.159	1.39E-01
203323_at	BF197655	*CAV2*	−0.363	5.11E-04	−0.094	3.82E-01
203554_x_at	NM_004219	*PTTG1*	0.402	1.05E-04	0.132	2.20E-01
207011_s_at	NM_002821	*PTK7*	0.265	1.26E-02	0.232	2.95E-02
207168_s_at	NM_004893	*H2AFY/H2AX*	0.411	7.03E-05	0.161	1.35E-01
207543_s_at	NM_000917	*P4HA1*	0.449	1.12E-05	0.424	3.89E-05
208680_at	L19184	*PRDX1*	0.258	1.51E-02	0.111	3.05E-01
208694_at	U47077	*PRKDC/DNA-PKcs*	0.409	7.64E-05	0.229	3.21E-02
208767_s_at	AW149681	*LAPTM4B*	0.329	1.75E-03	0.130	2.27E-01
209030_s_at	NM_014333	*CADM1/TSLC1*	0.196	6.70E-02	0.136	2.05E-01
209031_at	AL519710	*CADM1/TSLC1*	0.231	3.03E-02	0.143	1.85E-01
209543_s_at	M81104	*CD34*	−0.363	5.11E-04	−0.239	2.52E-02
210495_x_at	AF130095	*FN1*	0.286	6.99E-03	0.096	3.73E-01
210559_s_at	D88357	*CDK1/CDC2*	0.435	2.34E-05	0.259	1.50E-02
212097_at	AU147399	*CAV1*	−0.237	2.64E-02	−0.163	1.28E-01
212464_s_at	X02761	*FN1*	0.286	6.99E-03	0.0944	3.82E-01
217294_s_at	U88968	*ENO1/MBP1*	0.387	1.97E-04	0.187	8.03E-02
217871_s_at	NM_002415	*MIF*	0.421	4.41E-05	0.308	3.47E-03
218308_at	NM_006342	*TACC3*	0.333	1.52E-03	0.136	2.05E-01
218502_s_at	NM_014112	*TRPS1*	0.276	9.23E-03	0.242	2.31E-02
218755_at	NM_005733	*KIF20A/MKlp2*	0.407	8.35E-05	0.162	1.31E-01
219918_s_at	NM_018123	*ASPM*	0.399	1.16E-04	0.204	5.71E-02
220942_x_at	NM_014367	*FAM162A/HGTD-P*	0.151	1.60E-01	0.239	2.47E-02

### Hierarchical clustering based on the gene expression pattern of the 25 selected genes

**Figure 3:**
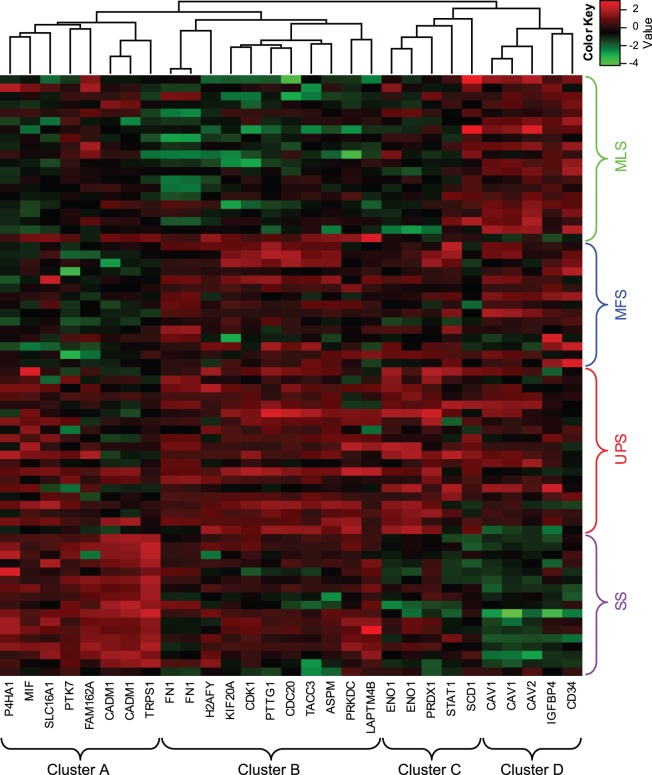
Heatmap and hierarchical clustering analyses. Twenty-nine probe sets were extracted using a simulation based on the permutation test (with adjusted *p*<0.05). The 29 probe sets were roughly divided into 4 clusters (clusters A–D). Columns represent probe sets, and rows represent samples. Red and green indicate high and low expression, respectively. UPS: undifferentiated pleomorphic sarcoma, MLS: myxoid liposarcoma, SS: synovial sarcoma, MFS: myxofibrosarcoma.

We performed hierarchical clustering for the 29 selected probe sets, representing 25 genes and 4 histological subtypes (UPS, MFS, MLS, and SS), as shown in Fig. 3. The genes were roughly classified into 4 clusters (clusters A, B, C, and D). Almost all genes were upregulated in both UPS and MFS. In addition, genes in cluster A were upregulated in SS, and genes in cluster D were upregulated in MLS.

### Analysis of the distribution of histological subtypes based on gene expression levels

**Figure 4:**
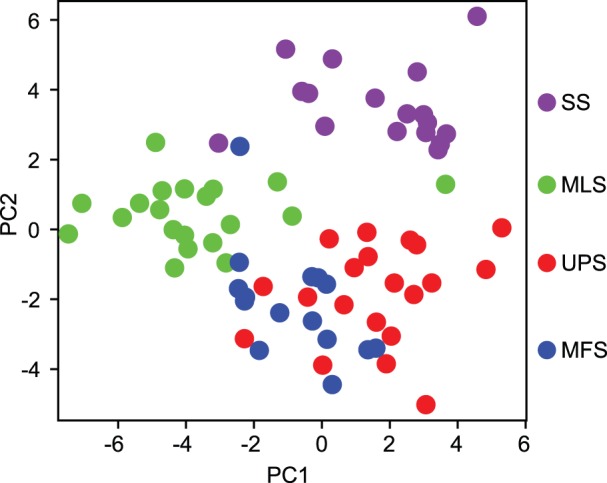
Principal component analysis using 29 probe sets for 4 histological types. The x-axis and y-axis represent the first and second principal components (PC1 and PC2), respectively. Each dot represents a sample colored according to its histological type. UPS: undifferentiated pleomorphic sarcoma, MLS: myxoid liposarcoma, SS: synovial sarcoma, MFS: myxofibrosarcoma.

**Figure 5:**
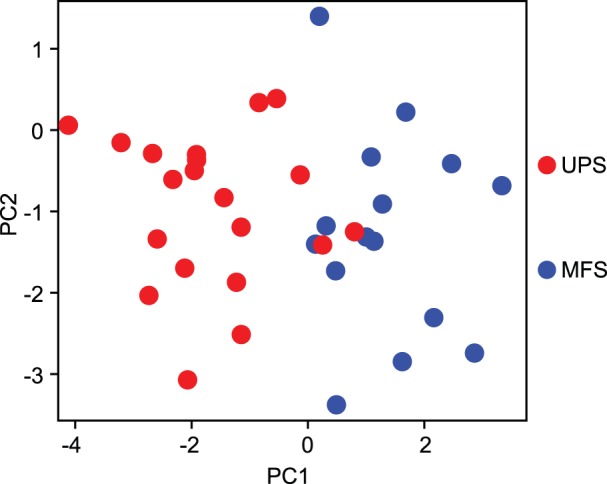
Principal component analysis using 9 probe sets for UPS and MFS. The x-axis and y-axis represent the first and second principal components (PC1 and PC2), respectively. Each dot represents a sample colored according to its histological type. UPS: undifferentiated pleomorphic sarcoma, MLS: myxoid liposarcoma, SS: synovial sarcoma, MFS: myxofibrosarcoma.

We performed PCA to calculate the first and the second PCs using the 29 probe sets. Detailed information on PCA, including eigenvector, standard deviation, proportion of variance, and cumulative proportion, is provided in Tables S4 and S5. The distribution of the 4 histological subtypes of STS on the 2 axes is shown in Fig. 4. The 4 histological subtypes were clearly classified into 3 clusters (SS, MLS, and UPS+MFS). This result indicated that UPS and MFS had histological similarities and similar gene expression patterns. Therefore, to discriminate between UPS and MFS, we applied Welch’s *t* test and the BH method to the gene expression data of the 29 probe sets, as shown in Table 4. We extracted 9 probe sets, representing 8 genes (*q* value <0.05): enolase 1 (*ENO1*)/c-myc-promoter binding protein-1 (*MBP1*); prolyl 4-hydroxylase subunit alpha-1 (*P4HA1*); peroxiredoxin 1 (*PRDX1*); *CD34*; family with sequence similarity 162, member A (*FAM162A*)/human growth and transformation-dependent protein (*HGTD-P*); protein tyrosine kinase 7 (*PTK7*); and macrophage migration inhibitory factor (*MIF*). We performed PCA to calculate the first and the second PCs from these 9 probe sets. Detailed information of PCA, including eigenvector, standard deviation, proportion of variance, and cumulative proportion, are shown in Table S5. The distribution of the 2 histological subtypes, UPS and MFS, on the 2 axes is shown in Fig. 5. UPS and MFS were classified into approximately 2 clusters. For the contribution of this classification, *MIF*, *ENO1*/*MBP1*, and *CD34* contributed to the top 3 largest coefficients for PC1, *PTK7*, *PRDX1*, and *ENO1*/*MBP1* contributed to the top 3 largest coefficients for PC2, and only *SCD1* contributed to the largest coefficients for PC3, as shown in Table S5. *MIF*, *ENO1*/*MBP1*, and *SCD1* were extracted in our previous study [51]. We also applied Welch’s *t* test and the BH method to the gene expression data from the 29 probe sets to discriminate UPS from SS and UPS from MLS, as shown in Table 4.

**Table 4:** Pairwise comparison between histological types using Welch’s t test for 29 probe sets.

Affymetrixprobe ID	Accessionno.	Gene symbol	UPS vs. MFS			UPS vs. SS			UPS vs. MLS		
			*p* value		*q* value	*p* value		*q* value	*p* value		*q* value
200832_s_at	AB032261	*SCD1*	7.36E-05	*	8.87E-04	1.06E-03	*	2.56E-03	3.52E-01		4.26E-01
200887_s_at	NM_007315	*STAT1*	2.81E-01		4.07E-01	1.54E-03	*	3.19E-03	2.04E-01		2.69E-01
201231_s_at	NM_001428	*ENO1/MBP1*	1.06E-04	*	8.87E-04	4.73E-08	*	6.85E-07	4.27E-06	*	1.42E-05
201508_at	NM_001552	*IGFBP4*	4.21E-02		1.15E-01	7.39E-03	*	1.13E-02	7.25E-02		1.00E-01
202236_s_at	NM_003051	*SLC16A1/MCT1*	1.54E-01		2.80E-01	3.92E-01		4.06E-01	6.49E-04	*	1.25E-03
202870_s_at	NM_001255	*CDC20*	2.10E-01		3.58E-01	1.23E-03	*	2.74E-03	6.26E-06	*	1.78E-05
203065_s_at	NM_001753	*CAV1*	8.76E-01		8.76E-01	5.56E-07	*	2.69E-06	5.31E-01		5.93E-01
203323_at	BF197655	*CAV2*	8.45E-01		8.75E-01	6.14E-05	*	1.98E-04	1.26E-03	*	2.15E-03
203554_x_at	NM_004219	*PTTG1*	3.76E-01		4.96E-01	8.95E-05	*	2.60E-04	1.59E-08	*	2.31E-07
207011_s_at	NM_002821	*PTK7*	6.14E-03	*	2.23E-02	4.21E-03	*	6.78E-03	9.19E-01		9.19E-01
207168_s_at	NM_004893	*H2AFY/H2AX*	4.37E-02		1.15E-01	1.18E-01		1.37E-01	6.75E-06	*	1.78E-05
207543_s_at	NM_000917	*P4HA1*	1.22E-04	*	8.87E-04	2.64E-02	*	3.48E-02	2.51E-03	*	4.05E-03
208680_at	L19184	*PRDX1*	1.84E-03	*	7.61E-03	5.31E-05	*	1.93E-04	1.36E-08	*	2.31E-07
208694_at	U47077	*PRKDC/DNA-PKcs*	5.49E-02		1.33E-01	9.76E-01		9.76E-01	1.13E-03	*	2.06E-03
208767_s_at	AW149681	*LAPTM4B*	4.20E-01		5.30E-01	3.73E-02	*	4.60E-02	8.30E-03	*	1.27E-02
209030_s_at	NM_014333	*CADM1/TSLC1*	2.49E-01		3.80E-01	2.81E-07	*	1.82E-06	6.43E-01		6.66E-01
209031_at	AL519710	*CADM1/TSLC1*	6.04E-02		1.35E-01	2.67E-07	*	1.82E-06	2.71E-01		3.42E-01
209543_s_at	M81104	*CD34*	8.73E-03	*	2.81E-02	1.78E-01		1.91E-01	3.97E-05	*	8.22E-05
210495_x_at	AF130095	*FN1*	4.83E-01		5.61E-01	2.50E-03	*	4.27E-03	3.53E-06	*	1.42E-05
210559_s_at	D88357	*CDK1/CDC2*	7.05E-02		1.46E-01	2.35E-02	*	3.24E-02	3.57E-06	*	1.42E-05
212097_at	AU147399	*CAV1*	6.43E-01		6.91E-01	3.14E-07	*	1.82E-06	4.16E-01		4.83E-01
212464_s_at	X02761	*FN1*	5.22E-01		5.83E-01	2.33E-03	*	4.22E-03	2.07E-06	*	1.20E-05
217294_s_at	U88968	*ENO1/MBP1*	4.24E-04	*	2.46E-03	4.07E-05	*	1.69E-04	1.55E-07	*	1.50E-06
217871_s_at	NM_002415	*MIF*	5.31E-06	*	1.54E-04	1.38E-01		1.54E-01	1.35E-05	*	3.27E-05
218308_at	NM_006342	*TACC3*	2.36E-01		3.80E-01	7.67E-04	*	2.02E-03	2.91E-05	*	6.49E-05
218502_s_at	NM_014112	*TRPS1*	3.64E-01		4.96E-01	5.21E-11	*	1.51E-09	1.85E-02	*	2.68E-02
218755_at	NM_005733	*KIF20A/MKlp2*	4.44E-01		5.37E-01	9.97E-03	*	1.45E-02	4.41E-06	*	1.42E-05
219918_s_at	NM_018123	*ASPM*	1.11E-01		2.15E-01	2.25E-03	*	4.22E-03	7.89E-07	*	5.72E-06
220942_x_at	NM_014367	*FAM162A/HGTD-P*	1.39E-03	*	6.70E-03	3.81E-02	*	4.60E-02	6.23E-01		6.66E-01

*q <0.05. The *p* value was calculated using Welch’s t test, and the *q* value was calculated from the *p* value by means of the Benjamini-Hochberg method for the correction of multiple testing problems.

### Classification of the 25 genes based on pairwise comparison of histological subtypes

**Figure 6:**
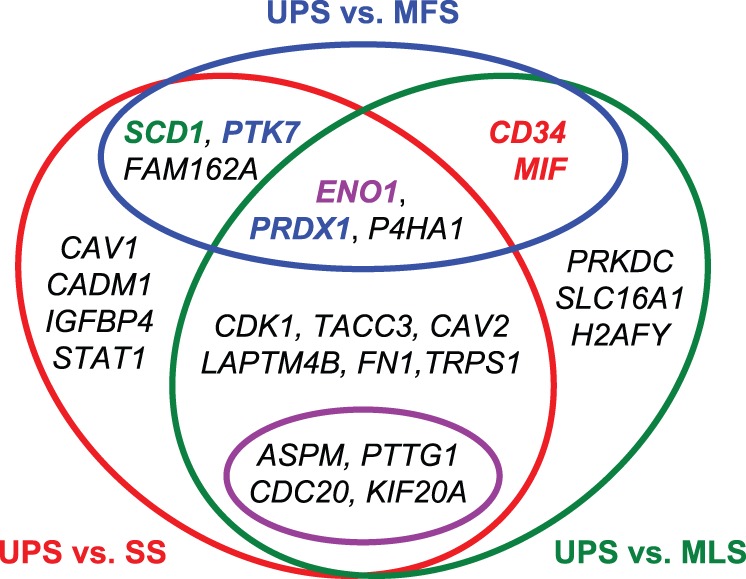
A Venn diagram of gene classification based on pairwise comparisons of histological types using Welch’s *t* test. Genes inside the red circle were statistically significant (*q* <0.05 calculated using Welch’s *t* test and the BH method) in the comparison of UPS with SS. Genes inside the green oval were statistically significant (*q* <0.05) in the comparison of UPS with MLS. Genes inside the blue oval were statistically significant (*q* <0.05) in the comparison of UPS and MFS. Genes inside the pink oval are common to CINSARC and our 25-gene set. For PCA of the 9-probe set, *MIF* and *CD34* highlighted in red were the first and third largest contributing coefficients to PC1, respectively. *PTK7* and *PRDX1* highlighted in blue were the first and second largest contributing coefficients to PC2, respectively. *ENO1*/*MBP1* highlighted in purple was the second largest contributing coefficient to PC1 and the third largest contributing coefficient to PC2. *SCD1* highlighted in green was the largest contributing coefficient to PC3.

We classified the 25 genes into 7 groups on the basis of 3 comparisons (UPS vs. MFS, UPS vs. SS, and UPS vs. MLS), as shown in Fig. 6. Only 3 genes, *ENO1/MBP1*, *P4HA1*, and *PRDX1*, were commonly selected (genes that were selected in the UPS vs. MFS comparison were also selected in the UPS vs. SS or UPS vs. MLS comparison). Furthermore, we compared the 25 genes selected in our study with the genes involved in the complexity index in sarcomas (CINSARC) [59] because the use of CINSARC (composed of 67 genes) instead of the FNCLCC grading system [1], [2] was recently proposed for predicting metastasis in STS [59]. In this comparison, only 4 common genes, that is, pituitary tumor-transforming 1 (*PTTG1*), abnormal spindle-like microcephaly-associated protein (*ASPM*), cell-division cycle protein 20 (*CDC20*), and kinesin family member 20A (*KIF20A*)/mitotic kinesin-like protein 2 (*MKlp2*), were extracted. The differential expression of these 4 genes was statistically significant (*q* <0.05) for UPS vs. SS and for UPS vs. MLS, but not for UPS vs. MFS. These 4 genes belonged to cluster B, as shown in Fig. 3. Consequently, the 25 genes were classified into 7 groups on the basis of pairwise comparisons of histological subtypes, as shown in Fig. 4.

### Survival analysis in UPS patients

**Figure 7:**
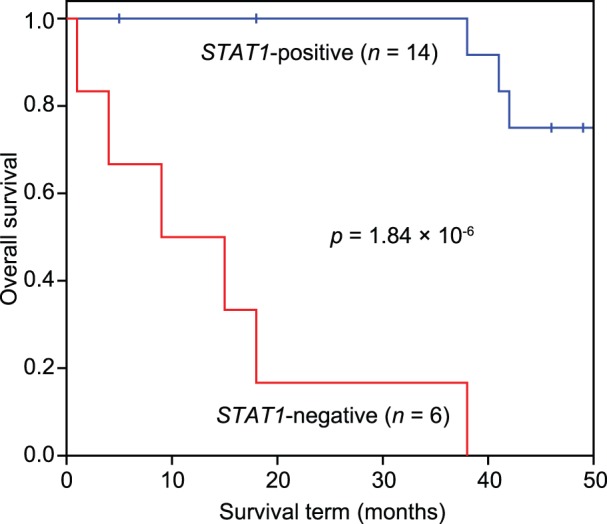
The Kaplan-Meier curve and the logrank test for *STAT1* in UPS patients. The *STAT1*-positive group (*STAT1* expression level >4871.5) consisted of 14 patients (blue line), and the *STAT1*-negative group consisted of 6 patients (red line). A hazard ratio (exp(B) = 30.2) was calculated using the Cox proportional hazards model.

We used the logrank test to analyze the survival of UPS patients. We selected the best *p* value for various thresholds (30th, 40th, 50th, 60th, 70th, and 80th percentiles) of gene expression signals in UPS patients for each probe set when the gene expression signals were binarized. Adjusted *p* values were obtained by adjusting the data for the multiple testing problem (6 thresholds×29 probe sets) based on the permutation test, as shown in Table S6. Only *STAT1* showed a statistically significant association with survival in UPS (logrank *p* value 1.84×10^−6^ and adjusted *p* value 2.99×10^−3^ after the permutation test). Fig. 7 shows that *STAT1-*positive and *STAT1*-negative groups had clearly different survival curves based on the Kaplan-Meier method.

## Discussion

In the present study, we conducted a simulation based on a permutation test to extract genes that are both diagnostic markers (for discrimination of histological subtypes) and prognostic markers (for overall survival in STS). As shown in Table 2, 25 genes were extracted, and their adjusted *p* values were statistically significant (adjusted *p*<0.05). We analyzed studies related to these 25 genes and found many reports suggesting that these 25 genes are effective prognostic/predictive factors or therapeutic targets, as shown in Table S7, according to the literature (See Supplementary Discussion).

**Figure 8:**
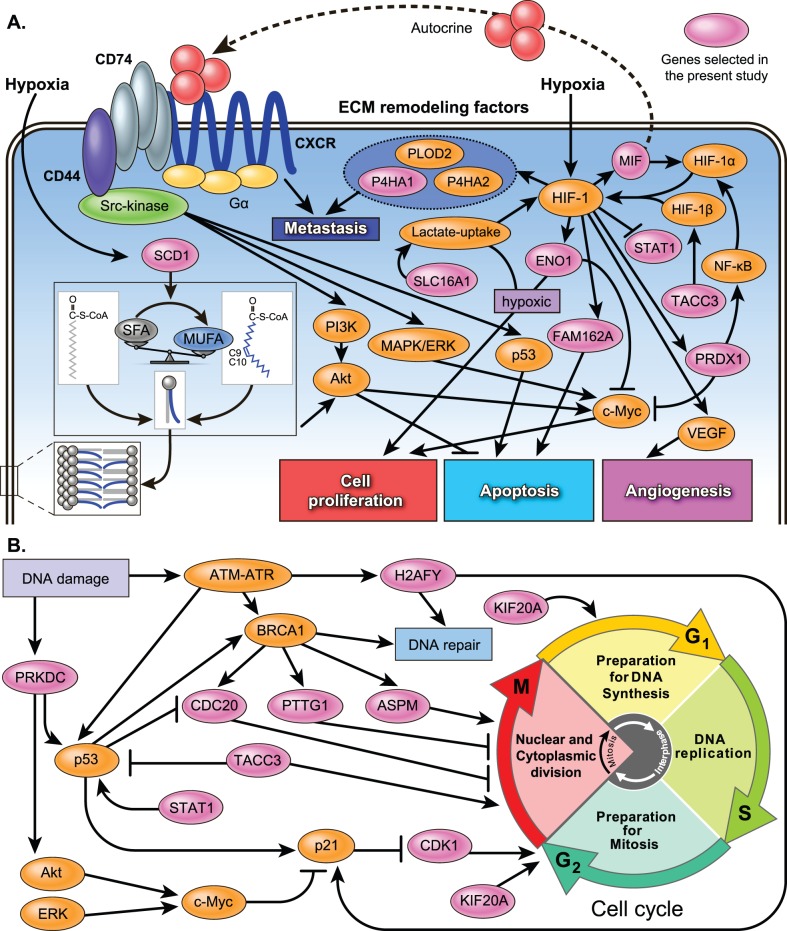
A hypothetical regulation model of metabolic and signaling control in highly malignant STS. (A) Signaling pathways, excluding cell cycle and DNA repair. (B) Cell cycle and DNA repair pathways. The pink oval indicates the genes selected in the present study. MUFA, monounsaturated fatty acid; SFA, saturated fatty acid; SCD1, stearoyl-CoA desaturase 1; MIF, macrophage migration inhibitory factor; CXCR, CXC chemokine receptor; PI3K, phosphoinositide 3-kinase; MAPK, extracellular signal-regulated kinase; ERK, mitogen-activated protein kinase; PTTG1, pituitary tumor-transforming 1; ASPM, abnormal spindle-like microcephaly-associated protein; CDC20, cell division cycle protein 20; KIF20A, kinesin family member 20A; ENO1, enolase 1; P4HA, prolyl 4-hydroxylase subunit α; PRDX1, peroxiredoxin 1; FAM162A, family with sequence similarity 162, member A; STAT1, signal transducer and activator of transcription 1; CDK1, cyclin-dependent kinase 1; TACC3, transforming, acidic coiled-coil containing protein 3; PRKDC, protein kinase, DNA-activated, catalytic polypeptide; H2AFY, H2A histone family, member Y; SLC16A1, solute carrier family 16, member 1; VEGF, vascular endothelial growth factor; HIF, hypoxia inducible factor; PLOD2, procollagen-lysine,2-oxoglutarate 5-dioxygenase 2; NF-κB, nuclear factor-kappa B.

Although we did not try to identify the molecular mechanisms behind the 25 selected genes, several published studies have examined pathways related to these 25 genes, as shown in Table S7 and Fig. 8. These 25 genes are roughly classified into 4 types, namely, hypoxia-related genes (*MIF*, *SCD1*, *P4HA1*, *ENO1*/*MBP1*, *FAM162A*/*HGTD-P*, *SLC16A1*/*MCT1*, *FN1*, and *STAT1*), cell cycle- and DNA repair-related genes (*ASPM*, *CDK1*/*CDC2*, *CDC20*, *KIF20A*/*MKlp2*, *PTTG1*, *TACC3*, *PRDX1*, *PRKDC*/*DNA-PKcs*, and *H2AFY*/*H2AX*), growth factor signal transduction-related genes, and other genes. Cell cycle- and DNA repair-related genes, hypoxia-induced genes, and growth factor signal transduction-related genes are key players in tumor growth, angiogenesis, metabolism, invasion, and metastasis in various types of cancer. In fact, these processes are attenuated by the inhibition or silencing of many of these 25 genes, as shown in Table S7. These genes are therefore possible prognostic/predictive markers and/or therapeutic targets.

*STAT1* expression was found to be strongly associated with survival in UPS patients. STAT1 interacts directly with p53 and induces cell growth arrest and apoptosis, as shown in Fig. 8. Although *STAT1* is repressed by HIF-1, the *STAT1*-positive group among the UPS patients had a better prognosis, even when hypoxia-related genes were upregulated. Therefore, *STAT1* is a possible novel, independent prognostic/predictive factor of STS, particularly UPS.

In the diagnosis of STS, classification of UPS is the most controversial topic. Among the 25 selected genes, hypoxia-related genes (*MIF*, *SCD1*, *P4HA1*, *ENO1*/*MBP1*, *FAM162A*/*HGTD-P*, *SLC16A1*/*MCT1*, *FN1*, and *STAT1*) are present in this study. In particular, the genes *MIF*, *SCD1*, *P4HA1*, *ENO1*/*MBP1*, and *FAM162A*/*HGTD-P* are differentially expressed between UPS and MFS, as shown in Fig. 6 and Table 4. Furthermore, *STAT1* is a prognostic marker in UPS patients, as shown in Fig. 7. Therefore, these hypoxia-related genes are promising prognostic and therapeutic targets and, if validated, may improve the treatment/diagnosis of this type of cancer. Further research is needed regarding the hypoxia-related pathways in highly malignant STS.

We manually constructed a hypothetical regulation model (Figure 8) of metabolic and signaling control in highly malignant STS. Nevertheless, according to the literature, a part of these networks could be automatically predicted by pathway and interaction analyses. For example, pathways of the cell cycle and the DNA damage response were identified by IntPath [33], [60], [61] with statistical significance (*q* value <0.05), as shown in Table S8. Interaction networks of the cell cycle (*ASPM*, *CDK1*, *CDC20*, *KIF20A*, *PTTG1*, *PRKDC*, and *TACC3*) and *HIF-1* (*MIF*, *ENO1*, and *PRDX1*) were identified by means of STRING [62], as shown in Fig. S1. Nonetheless, these tools should be used with appropriate parameters [34], [60], [61]. Such tools are more effective methods when large numbers of candidate genes are extracted.

In summary, we analyzed microarray gene expression data from 88 STS patients using a combination method involving knowledge-based filtering and a simulation based on the integration of multiple statistics to reduce multiple testing problems. Our combination method automatically identified 25 genes in the gene expression data from STS. These genes showed significant differential expression between different histological subtypes, including UPS, and showed associations with survival in STS. Furthermore, we conducted a bibliographic survey in terms of cancer progression for the 25 identified genes, and substantial evidence was uncovered in the literature. These genes were roughly classified into 4 types, namely, hypoxia-related genes, cell cycle- and DNA repair-related genes, growth factor signal transduction-related genes, and other genes. *STAT1* showed a statistically significant association with the survival of UPS patients (logrank adjusted *p* = 0.00299). Although only a few studies have investigated the association of these genes with survival in STS, many recent studies have reported that these genes are prognostic factors and/or therapeutic targets in other types of cancers. Therefore, these results suggest that our combination method is capable of identifying genes that are potential prognostic/predictive factors and/or therapeutic targets in STS and possibly in other cancers. These disease-associated genes deserve further preclinical and clinical validation.

## Supporting Information

Figure S1 **The pathways predicted by STRING from the 25 selected genes.**
(PDF)

Table S1 **Clinical data of the 88 patients with soft tissue sarcoma.** UPS: undifferentiated pleomorphic sarcoma, MLS: myxoid liposarcoma, SS: synovial sarcoma, MFS: myxofibrosarcoma, LMS: leiomyosarcoma, FS: fibrosarcoma, MPNST: malignant peripheral nerve sheath tumor, Tumor metastasis indicates the incidence of tumor metastasis in STS patients.
(XLS)

Table S2 **The MIM number list.**
(XLS)

Table S3 **Selected Affymetrix probe IDs.**
(XLS)

Table S4 **Information on PCA, including the eigenvector, standard deviation, proportion of variance, and cumulative proportion for 29 probe sets.** PCA: principal component analysis, PC: principal components.
(XLS)

Table S5 **Information on PCA, including the eigenvector, standard deviation, proportion of variance, and cumulative proportion for 9 probe sets.** PCA: principal component analysis, PC: principal components.
(XLS)

Table S6 **Survival analysis in UPS using the logrank test.** Adjusted *p* values were calculated using the permutation test (100,000 repeats) from logrank *p* values.
(XLS)

Table S7 **Gene or pathway annotations and likelihood as prognostic/predictive factors and/or therapeutic targets.** Adjusted *p* values were calculated using the permutation test (100,000 repeats) from logrank *p* values.
(XLS)

Table S8 **Pathway analysis in IntPath.** *k*: genes from the overlap between genes in the list and genes in the pathway, *n*: the number of genes in the input gene list, *m*: the number of genes in the identified pathways, *N*: the total number of genes. The *p* values were calculated using the hypergeometric test; the *q* values were calculated from the *p* values using the Benjamini-Hochberg (BH) method.
(XLS)

Information S1 (PDF)
