# Supplementary figures and images for: Analysis of Gene Expression Profiles of Soft Tissue Sarcoma Using a Combination of Knowledge-Based Filtering with Integration of Multiple Statistics
Source: PLoS One. 2014 Sep 4;9(9):e106801. doi: 10.1371/journal.pone.0106801 (PMC4154757; doi:10.1371/journal.pone.0106801)

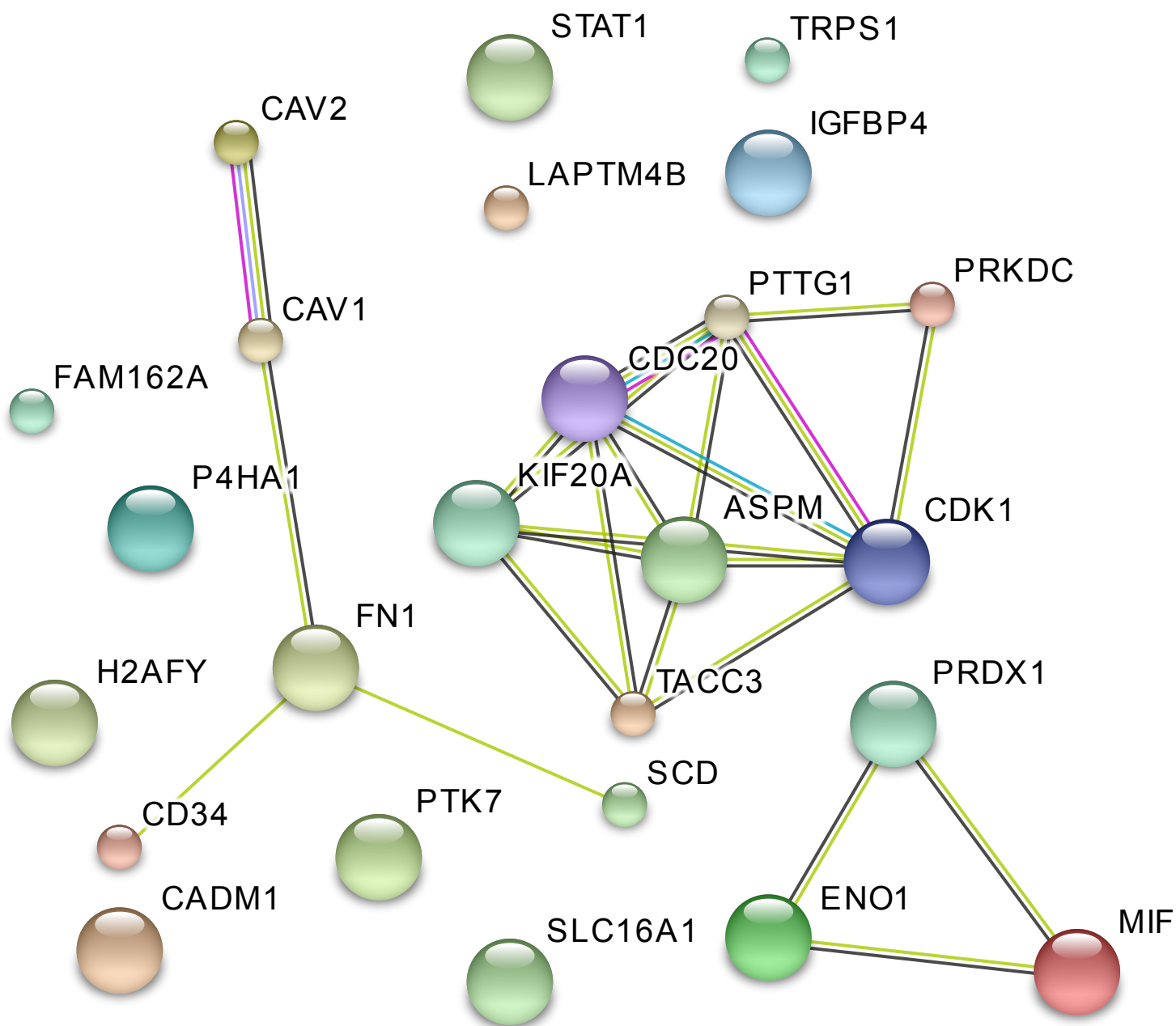

Supplement: Figure S1 — The pathways predicted by STRING from the 25 selected genes. (PDF) [file pone.0106801.s001.pdf]
