# Supplementary material for: Analysis of Gene Expression Profiles of Soft Tissue Sarcoma Using a Combination of Knowledge-Based Filtering with Integration of Multiple Statistics
Source: PLoS One. 2014 Sep 4;9(9):e106801. doi: 10.1371/journal.pone.0106801 (PMC4154757; doi:10.1371/journal.pone.0106801)
Supplement: Information S1 — (PDF) [file pone.0106801.s010.pdf]

Supplementary Information

Supplementary Discussion

In the present study, we conducted a simulation based on a permutation test to extract genes that are both diagnostic markers (for discrimination of histological subtypes) and prognostic markers (for overall survival in STS). As shown in Table 2, 25 genes were extracted, and their adjusted p values were statistically significant (adjusted $p < 0.05$). We analyzed studies related to these 25 genes and found many reports suggesting that these 25 genes are effective prognostic/predictive factors or therapeutic targets, as shown in Supplementary Table S7. Among the 25 genes, 8 genes were extracted using Welch's t test to compare UPS and MFS ($q < 0.05$). Four of these genes, *MIF*, *SCD1*, *ENO1/MBP1*, and *P4HA1*, were also extracted in our previous study [1]. Among the 4 genes, *MIF* and *SCD1* are potential prognostic/predictive markers and/or therapeutic targets not only for UPS but also for other cancers [2-26].

MIF expression is induced by HIF-1 under hypoxia [27,28]. Secreted MIF interacts with the cell surface molecule CD74 [29]. CD74 lacks a signal-transducing intracellular domain but interacts with the proteoglycan CD44 and mediates signaling via CD44 to activate Src-family kinase and mitogen-activated protein kinase (MAPK)/extracellular signal-regulated kinase (ERK), either to stimulate the phosphatidylinositol 3-kinase (PI3K)/Akt pathway or to initiate the p53-dependent inhibition of apoptosis [30]. MIF can also promote invasion and metastasis via G-protein-coupled chemokine receptors (CXCR2, CXCR4, and CXCR7) [22,31,32]. Furthermore, MIF activates *HIF-1α* expression in a p53-dependent manner [33]. MIF promotes not only tumor metastasis [1,12,19,21,22] but also apoptosis [34,35], cell growth [10,18], and angiogenesis [2,3,36]. *SCD1* expression is also induced by hypoxia [37]. *SCD1* and *HIF-2α* are overexpressed in clear cell renal cell

carcinoma under hypoxia; they synergistically inhibit apoptosis and promote cell migration [38]. Hypoxia-induced SCD1 activates the Akt pathway via regulation of the Δ9 monounsaturated fatty acid (MUFA)/saturated fatty acid (SFA) balance in mammalian cells [37,39,40].

The 2 remaining genes, *ENO1/MBP1* and *P4HA1*, also showed statistical significance, not only for UPS vs. MFS but also for UPS vs. SS and for UPS vs. MLS, as shown in Table 4 and Fig. 6. Recent studies have revealed that *ENO1/MBP1* and *P4HA1* are target genes of hypoxia-inducible factor 1 (*HIF-1*) [41,42]. Furthermore, *ENO1/MBP1* is a repressor of *MYC* [43], a prognostic marker for many cancers [44-49] and a therapeutic target in breast cancer (in combination with radiation therapy) [50]. Knockdown of *ENO1/MBP1* inhibits hypoxic cell growth in clear cell ovarian cancer [51]. Hypoxia-induced *P4HA1*, *P4HA2*, and procollagen-lysine,2-oxoglutarate 5-dioxygenase 2 (*PLOD2*) promote invasiveness and metastasis by altering the composition, alignment, and mechanical properties of the extracellular matrix (ECM) [41]. *P4HA1* and *P4HA2* encode collagen prolyl hydroxylases that are essential for cancer invasion and metastasis [52]. Among our 25 genes, *P4HA1* showed the strongest association with the histological grade ($\rho = 0.449$, $p = 1.12 \times 10^{-5}$) and with metastasis ($\rho = 0.424$, $p = 3.89 \times 10^{-5}$), as shown in Table 3. These results suggest that the malignancy of UPS depends on the activation of genes downstream of *HIF*s under hypoxic conditions. A study of the relationship between a hypoxia-induced transcription profile and the metastatic potential of STS has been published [53]. In the present study, we enriched the set of potential disease-associated genes by combining knowledge-based filtering with a simulation based on the integration of multiple statistics.

Among the 8 genes selected by Welch's *t* test (comparison of UPS with MFS), 4 genes, that is, *MIF*, *SCD1*, *ENO1*, and *P4HA1*, had been extracted in our previous study [1]. The 4 remaining genes, that is, *PRDX1*, *CD34*, *FAM162A*/*HGTD-P*, and *PTK7*, have not been

extracted by comparison of UPS with MFS in any previous study. CD34 has been used successfully for the differential diagnosis (immunohistochemical analysis) of STS [54]. CD34 is also a marker for progenitor and stem cells [55,56] and vascular endothelial cells [57]. Therefore, CD34 will likely be a useful prognostic marker in some cancers [58-62]. *PRDX1* encodes a member of the peroxiredoxin family of antioxidant enzymes. PRDX1 interacts with the cellular oncogene products c-Abl and c-Myc and inhibits c-Abl kinase activity [63] and Myc-mediated transformation [64], independent of its antioxidant activity. Thus, PRDX1 acts as a tumor suppressor. In addition, PRDX1 promotes nuclear factor kappa B (NF-κB) activity, which induces the expression of HIF-1α via toll-like receptor 4 (TLR4) in prostate cancer [65]. NF-κB directly binds to the *HIF-1α* promoter [66]. Many studies suggest that *PRDX1* is a useful prognostic marker in various cancers [67-71], but there are no data for STS in this regard. In the present study, *PRDX1* showed statistical significance not only for UPS vs. MFS but also for UPS vs. SS and UPS vs. MLS, as shown in Table 4 and Fig. 6.

FAM162A/*HGTD-P* is a HIF-1 target gene and is an indispensable mediator of the mitochondrial apoptotic pathway [72,73]. PTK7 is a regulator of noncanonical WNT/planar cell polarity (PCP) signaling [74]. PTK7 also inhibits canonical Wnt/β-catenin signaling [75,76]. *PTK7* is upregulated in many cancers, and knockdown of *PTK7* in colon carcinoma cells inhibits cell proliferation and induces caspase 10-dependent apoptosis via the mitochondrial pathway [77]. Many studies suggest that *PTK7* is a useful prognostic/predictive marker and/or therapeutic target in various cancers [77-84].

When CINSARC was compared with our 25 genes, 4 common genes, *PTTG1*, *ASPM*, *CDC20*, and *KIF20A*/*MKlp2*, were extracted, as shown in Fig. 4. *PTTG1*, *ASPM*, and *CDC20* are downstream of breast cancer susceptibility gene 1 (*BRCA1*) [85]. Knockdown of *BRCA1* with small interfering RNA (siRNA) downregulates multiple genes implicated in

chromosome segregation (e.g., *PTTG1*), centrosome function (e.g., *ASPM*), and progression into and through mitosis (e.g., *CDC20*) in human prostate (DU-145) and breast (MCF-7) cancer cells [85]. *PTTG1* is a proto-oncogene originally cloned from a rat pituitary tumor [86]. *PTTG1* encodes a protein that interacts with p53, modulates p53-mediated transcriptional activity and apoptosis [87], and prevents the activation of separin, which induces sister chromatid separation in the transition from metaphase to anaphase [88]. Many studies suggest that *PTTG1* is a useful prognostic/predictive marker and/or therapeutic target in various cancers [89-98].

ASPM is the putative human ortholog of the *Drosophila melanogaster* abnormal spindle gene (Asp), which is involved in mitosis [99] and DNA repair [100]. Many studies suggest that *ASPM* is a useful prognostic/predictive marker and/or therapeutic target in various cancers [101-106]. CDC20 is an essential regulator of cell division in humans [107,108]. CDC20 is downregulated by p53 [109], and many studies have suggested that *CDC20* is a useful prognostic/predictive marker and/or therapeutic target in various cancers [110-116].

KIF20A/MKlp2, encodes a member of the kinesin superfamily of motor proteins [117]. KIF20A/MKlp2 is a mitotic inhibitory target of mitotic arrest deficient 2 (MAD2) and is necessary for proper mitotic progression and cytokinesis [118]. Direct interaction between MAD2 and CDC20 is a key event during the checkpoint activation of spindle assembly [119]. Inhibition of *KIF20A*/*MKlp2* induces lysosomal cell death and cell cycle arrest in the G1 phase in breast cancer cells [120] and G2/M arrest in gastric cancer cells [121]. Many studies suggest that *KIF20A*/*MKlp2* is a useful prognostic/predictive marker and/or therapeutic target in different types of cancer [120-123].

These results indicate that the 4 genes common to CINSARC and our analysis are linked to cell cycle checkpoints. This is logical because CINSARC genes were selected for

the prediction of metastasis in STS, and our 25 genes were selected as both diagnostic markers (for histological subtypes) and prognostic markers of overall survival in STS. Among the 25 genes, 13 genes remained after using Welch's *t* test to compare UPS and SS or UPS and MLS ($q < 0.05$): solute carrier family 16, member 1 (*SLC16A1*)/monocarboxylate transporter 1 (*MCT1*); cell adhesion molecule 1 (*CADM1*)/tumor suppressor in non-small cell lung cancer 1 (*TSLC1*); trichorhinophalangeal syndrome 1 (*TRPS1*); protein kinase, DNA-activated, catalytic polypeptide (*PRKDC*)/DNA-dependent protein kinase catalytic subunit (*DNA-PKcs*); cyclin-dependent kinase 1 (*CDK1*)/cell division cycle protein 2 (*CDC2*); transforming, acidic coiled-coil containing protein 3 (*TACC3*); lysosomal protein transmembrane 4 β (*LAPTM4B*); fibronectin 1 (*FN1*); H2A histone family, member Y (*H2AFY*)/histone H2A variant (*H2AX*); *STAT1*; caveolin 1 (*CAV1*); caveolin 2 (*CAV2*); and insulin-like growth factor-binding protein 4 (*IGFBP4*).

SLC16A1/*MCT1* encodes a monocarboxylate transporter that drives the movement of lactate and pyruvate across cell membranes [124]. SLC16A1/MCT1 counteracts p53 activity at the transcriptional level, and the loss of p53 along with SLC16A1/MCT1 overexpression synergistically promotes chromosomal instability and tumorigenicity [125]. Lactate taken up by SLC16A1/MCT1 activates HIF-1 and triggers tumor angiogenesis and tumor growth [126,127]. SLC16A1/MCT1 inhibition has antitumor effects that are associated with the NF-κB pathway [128]. Many studies suggest that *SLC16A1*/*MCT1* is a promising prognostic/predictive marker and/or therapeutic target in various types of cancer [126,128-135].

CADM1/*TSLC1* encodes an immunoglobulin-like cell adhesion molecule with 3 immunoglobulin loops [136]. The ectodomain of CADM1/TSLC1 mediates intercellular adhesion through homophilic or heterophilic *trans*-interactions between neighboring cells [137]. CADM1/TSLC1 is implicated in cell proliferation, invasion, and apoptosis via

regulation of the Akt signaling pathway [138]. Promoter methylation of the *CADM1*/*TSLC1* gene is frequently observed in many cancers [139]. CADM1/TSLC1 is a useful prognostic/predictive marker and/or therapeutic target because it acts as a tumor suppressor in various cancers [138,140-143]. In addition, T-cell lymphoma invasion and metastasis 1 (TIAM1) integrates signals from CADM1/TSLC1 to regulate the actin cytoskeleton through Rac activation, which leads to tissue infiltration by leukemic cells in adult T-cell leukemia/lymphoma (ATL) [136].

TRPS1 encodes a GATA-type zinc-finger protein [144]. TRPS1 is a nuclear protein that binds GATA sequences, and TRPS1 potently and specifically prevents transcriptional activation mediated by other GATA factors [144]. TRPS1 is a crucial regulator of the mesenchymal-to-epithelial cell transition [145,146]; accordingly, *TRPS1* is a prognostic marker in breast cancer [145,146] and colon cancer [147].

PRKDC/*DNA-PKcs* encodes a member of the phosphatidylinositol 3-kinase-like kinase (PIKK) family of protein kinases [148]. Ataxia telangiectasia mutated (ATM) and ataxia telangiectasia and Rad3-related (ATR) protein also belong to the PIKK family, and PRKDC/DNA-PKcs cooperates with these genes to phosphorylate proteins involved in the DNA damage checkpoint [149]. Inhibition of PRKDC/DNA-PKcs activity prevents binding of PRKDC/DNA-PKcs to p53 on the *p21* promoter [150]. Several studies suggest that *PRKDC*/*DNA-PKcs* is a good prognostic/predictive marker and/or therapeutic target in various cancers [151-153].

CDK1/CDC2 is a serine/threonine kinase that interacts with cyclin B1 (CCNB1) to form a complex known as the maturation-promoting factor (MPF), which is essential for cell cycle progression through mitosis [154]. Inhibition of CDK1/CDC2 induces G2/M arrest [155]. Many studies suggest that *CDK1*/*CDC2* is a useful prognostic/predictive marker and/or therapeutic target in various types of cancer [156-160].

TACC3 is necessary for the regulation of mitotic spindle assembly and chromosome segregation [161]. This protein regulates the transcriptional activation of HIF-1 through interaction with aryl hydrocarbon receptor nuclear translocator (ARNT) (also known as HIF-1β) [162]. TACC3 deficiency is associated with a high rate of apoptosis and expression of the p53 target gene *p21* [163]. Several studies suggest that *TACC3* is a useful prognostic/predictive marker and/or therapeutic target in various cancers [164-166].

LAPTM4B, which encodes a protein containing a lysosome localization motif that localizes to the late endosomes and lysosomes [167], was originally identified as a hepatocellular carcinoma-associated gene [167]. LAPTM4B promotes autophagy and tolerance to metabolic stress [168], enhances the multidrug resistance of cancer cells by promoting drug efflux through colocalization and interaction with P glycoprotein (P-gp), and inhibits apoptosis by activating PI3K/AKT signaling [169]. Many studies suggest that *LAPTM4B* is a useful prognostic/predictive marker and/or therapeutic target in various cancers [170-174].

FN1, a HIF-1 target gene in colon carcinoma [175] and a SRY-related HMG-box gene 2 (*SOX2*) target gene in ovarian cancer [176], promotes cell migration and invasion in various cancers [177,178].

H2AFY/*H2AX* encodes a histone protein that consists of a histone H2A-like histone domain and a large, globular C-terminal macrodomain that is not present in other histone proteins [179]. H2AFY/H2AX is required for the repair of DNA double-strand breaks in the ATM signaling pathway [180], and active expression of H2AFY/H2AX (γH2AX) is an indicator of DNA double-strand breaks [181]. H2AFY/H2AX was found to interact with human epidermal growth factor receptor 2 (HER2) in cancer cells that overexpress HER2 [182]. H2AFY/H2AX has also been shown to interact with tumor suppressor p53-binding protein 1 (TP53BP1) [183-185], BRCA1 [186-188], and BRCA1-associated RING domain

protein 1 (BARD1) [186,188]. The absence of H2AFY/H2AX causes proteasome-dependent degradation of p21 under conditions of DNA damage [189]. Many studies have suggested that *H2AFY*/*H2AX* is a promising prognostic marker in various cancers [190-193].

The protein encoded by *STAT1* was the first identified member of a multigene family targeted by both the type I and type II interferon (IFN) pathways [194-197]. STAT1 maintains cellular homeostasis by controlling cell growth, proliferation, apoptosis, and immune reactions. Expression and posttranslational aberrations of STAT1 have been identified in a variety of human pathological conditions, including cancer [198]. STAT1 acetylation depends on the balance between the activity of histone deacetylases (HDACs) and histone acetyltransferases (HATs). STAT1 acetylation is involved in the regulation of NF-κB activity and thus of apoptosis [199]. STAT1 also directly interacts with p53 to enhance DNA damage-induced apoptosis [200]. In addition, *STAT1* is downstream of HIF-1; the latter downregulates the expression of *STAT1* through differentiated embryo-chondrocyte expressed gene 1 (DEC1)/stimulated by retinoic acid 13 (STRA13) in an HDAC1-dependent manner [201]. Many studies have suggested that *STAT1* would be an effective prognostic/predictive marker and/or therapeutic target in various types of cancer [202-207].

CAV1 and CAV2 are integral membrane proteins that are essential components of caveolar membranes. They contribute to the negative regulation of tyrosine and serine/threonine kinase activities by binding to epidermal growth factor receptor (EGFR) [208]. CAV1 and CAV2 modulate downstream signaling, such as RAS/ERK signaling, via a nonreceptor tyrosine kinase (Src/Fyn/Abl) [209-211]. CAV2 interacts with CAV1 [210], and *CAV1* expression induces the downregulation of MAPK, PI3K/AKT, and mTOR signaling as well as the activation of apoptotic pathways [212]. Many studies have suggested that *CAV1* and *CAV2* are useful prognostic markers in various cancers [211-215]. However, these

genes might behave either as tumor suppressors or as oncogenes depending on the cell type and tumor stage/grade [216,217]. In the present study, *CAV1* and *CAV2* expression levels were negatively associated with the histological grade in STS, as shown in Table 3.

IGFBP4 encodes a protein that is a potent inhibitor of IGF activity [218-220]. IGFBP4 inhibits IGF-dependent growth and angiogenic effects in glioblastoma [221] and colorectal cancer [222]. In addition, IGFBP4 expression activates cell growth, metastasis, and Wnt/β-catenin signaling in renal cell carcinoma [223]. The expression of IGFBP4 in lung adenocarcinomas is downregulated by epigenetic silencing in association with tumor differentiation, resulting in the disruption of IGFBP4-mediated growth inhibition [224]. A negative correlation between the Ki-67 labeling index and IGFBP4 expression was reported in lung adenocarcinoma [224]. This result is consistent with our study results, as shown in Table 3.

Supplementary References

1. Takahashi H, Nakayama R, Hayashi S, Nemoto T, Murase Y, et al. (2013) Macrophage migration inhibitory factor and stearoyl-CoA desaturase 1: potential prognostic markers for soft tissue sarcomas based on bioinformatics analyses. PLoS One 8: e78250.

2. Cheng RJ, Deng WG, Niu CB, Li YY, Fu Y (2011) Expression of macrophage migration inhibitory factor and CD74 in cervical squamous cell carcinoma. Int J Gynecol Cancer 21: 1004-1012.

3. Choudhary S, Hegde P, Pruitt JR, Sielecki TM, Choudhary D, et al. (2013) Macrophage migratory inhibitory factor promotes bladder cancer progression via increasing proliferation and angiogenesis. Carcinogenesis 34: 2891-2899.

4. Du X, Wang QR, Chan E, Merchant M, Liu J, et al. (2012) FGFR3 stimulates stearoyl CoA desaturase 1 activity to promote bladder tumor growth. Cancer Res 72: 5843-5855.

5. Fenner A (2013) Kidney cancer: Stearoyl-CoA desaturase: a novel therapeutic target for RCC. Nat Rev Urol 10: 370.

6. Han I, Lee MR, Nam KW, Oh JH, Moon KC, et al. (2008) Expression of macrophage migration inhibitory factor relates to survival in high-grade osteosarcoma. Clin Orthop Relat Res 466: 2107-2113.

7. Leung JY, Kim WY (2013) Stearoyl co-A desaturase 1 as a ccRCC therapeutic target: death by stress. Clin Cancer Res 19: 3111-3113.

8. Mason P, Liang B, Li L, Fremgen T, Murphy E, et al. (2012) SCD1 inhibition causes cancer cell death by depleting mono-unsaturated fatty acids. PLoS One 7: e33823.

9. Meyer-Siegler KL, Iczkowski KA, Leng L, Bucala R, Vera PL (2006) Inhibition of macrophage migration inhibitory factor or its receptor (CD74) attenuates growth and invasion of DU-145 prostate cancer cells. J Immunol 177: 8730-8739.

10. Mittelbronn M, Platten M, Zeiner P, Dombrowski Y, Frank B, et al. (2011) Macrophage migration inhibitory factor (MIF) expression in human malignant gliomas contributes to immune escape and tumour progression. Acta Neuropathol 122: 353-365.

11. Noto A, Raffa S, De Vitis C, Roscilli G, Malpicci D, et al. (2013) Stearoyl-CoA desaturase-1 is a key factor for lung cancer-initiating cells. Cell Death Dis 4: e947.

12. Ren Y, Chan HM, Fan J, Xie Y, Chen YX, et al. (2006) Inhibition of tumor growth and metastasis in vitro and in vivo by targeting macrophage migration inhibitory factor in human neuroblastoma. Oncogene 25: 3501-3508.

13. Rendon BE, Roger T, Teneng I, Zhao M, Al-Abed Y, et al. (2007) Regulation of human lung adenocarcinoma cell migration and invasion by macrophage migration inhibitory factor. J Biol Chem 282: 29910-29918.

14. Roongta UV, Pabalan JG, Wang X, Ryseck RP, Fargnoli J, et al. (2011) Cancer cell dependence on unsaturated fatty acids implicates stearoyl-CoA desaturase as a target for cancer therapy. Mol Cancer Res 9: 1551-1561.

15. Scaglia N, Chisholm JW, Igal RA (2009) Inhibition of stearoylCoA desaturase-1 inactivates acetyl-CoA carboxylase and impairs proliferation in cancer cells: role of AMPK. PLoS One 4: e6812.

16. Scaglia N, Igal RA (2008) Inhibition of Stearoyl-CoA Desaturase 1 expression in human lung adenocarcinoma cells impairs tumorigenesis. Int J Oncol 33: 839-850.

17. Schrader J, Deuster O, Rinn B, Schulz M, Kautz A, et al. (2009) Restoration of contact inhibition in human glioblastoma cell lines after MIF knockdown. BMC Cancer 9: 464.

18. Schulz R, Marchenko ND, Holembowski L, Fingerle-Rowson G, Pesic M, et al. (2012) Inhibiting the HSP90 chaperone destabilizes macrophage migration inhibitory factor and thereby inhibits breast tumor progression. J Exp Med 209: 275-289.

19. Simpson KD, Templeton DJ, Cross JV (2012) Macrophage migration inhibitory factor promotes tumor growth and metastasis by inducing myeloid-derived suppressor cells in the tumor microenvironment. J Immunol 189: 5533-5540.

20. Sun B, Nishihira J, Suzuki M, Fukushima N, Ishibashi T, et al. (2003) Induction of macrophage migration inhibitory factor by lysophosphatidic acid: relevance to tumor growth and angiogenesis. Int J Mol Med 12: 633-641.

21. Sun B, Nishihira J, Yoshiki T, Kondo M, Sato Y, et al. (2005) Macrophage migration inhibitory factor promotes tumor invasion and metastasis via the Rho-dependent pathway. Clin Cancer Res 11: 1050-1058.

22. Tarnowski M, Grymula K, Liu R, Tarnowska J, Drukala J, et al. (2010) Macrophage migration inhibitory factor is secreted by rhabdomyosarcoma cells, modulates tumor metastasis by binding to CXCR4 and CXCR7 receptors and inhibits recruitment of cancer-associated fibroblasts. Mol Cancer Res 8: 1328-1343.

23. von Roemeling CA, Marlow LA, Wei JJ, Cooper SJ, Caulfield TR, et al. (2013) Stearoyl-CoA desaturase 1 is a novel molecular therapeutic target for clear cell renal cell carcinoma. Clin Cancer Res 19: 2368-2380.

24. Wang XB, Tian XY, Li Y, Li B, Li Z (2012) Elevated expression of macrophage migration inhibitory factor correlates with tumor recurrence and poor prognosis of patients with gliomas. J Neurooncol 106: 43-51.

25. Tomiyasu M, Yoshino I, Suemitsu R, Okamoto T, Sugimachi K (2002) Quantification of macrophage migration inhibitory factor mRNA expression in non-small cell lung cancer tissues and its clinical significance. Clin Cancer Res 8: 3755-3760.

26. Holder AM, Gonzalez-Angulo AM, Chen H, Akcakanat A, Do KA, et al. (2013) High stearoyl-CoA desaturase 1 expression is associated with shorter survival in breast cancer patients. Breast Cancer Res Treat 137: 319-327.

27. Welford SM, Bedogni B, Gradin K, Poellinger L, Broome Powell M, et al. (2006) HIF1alpha delays premature senescence through the activation of MIF. Genes Dev 20: 3366-3371.

28. Baugh JA, Gantier M, Li L, Byrne A, Buckley A, et al. (2006) Dual regulation of macrophage migration inhibitory factor (MIF) expression in hypoxia by CREB and HIF-1. Biochem Biophys Res Commun 347: 895-903.

29. Morand EF, Leech M, Bernhagen J (2006) MIF: a new cytokine link between rheumatoid arthritis and atherosclerosis. Nat Rev Drug Discov 5: 399-410.

30. Zernecke A, Bernhagen J, Weber C (2008) Macrophage migration inhibitory factor in cardiovascular disease. Circulation 117: 1594-1602.

31. Dessein AF, Stechly L, Jonckheere N, Dumont P, Monte D, et al. (2010) Autocrine induction of invasive and metastatic phenotypes by the MIF-CXCR4 axis in drug-resistant human colon cancer cells. Cancer Res 70: 4644-4654.

32. Bernhagen J, Krohn R, Lue H, Gregory JL, Zernecke A, et al. (2007) MIF is a noncognate ligand of CXC chemokine receptors in inflammatory and atherogenic cell recruitment. Nat Med 13: 587-596.

33. Oda S, Oda T, Nishi K, Takabuchi S, Wakamatsu T, et al. (2008) Macrophage migration inhibitory factor activates hypoxia-inducible factor in a p53-dependent manner. PLoS One 3: e2215.

34. Guo Y, Hou J, Luo Y, Wang D (2013) Functional disruption of macrophage migration inhibitory factor (MIF) suppresses proliferation of human H460 lung cancer cells by caspase-dependent apoptosis. Cancer Cell Int 13: 28.

35. Fersching DM, Nagel D, Siegele B, Salat C, Heinemann V, et al. (2012) Apoptosis-related biomarkers sFAS, MIF, ICAM-1 and PAI-1 in serum of breast cancer patients undergoing neoadjuvant chemotherapy. Anticancer Res 32: 2047-2058.

36. Giannice R, Erreni M, Allavena P, Buscaglia M, Tozzi R (2013) Chemokines mRNA expression in relation to the Macrophage Migration Inhibitory Factor (MIF) mRNA and Vascular Endothelial Growth Factor (VEGF) mRNA expression in the microenvironment of endometrial cancer tissue and normal endometrium: a pilot study. Cytokine 64: 509-515.

37. Li J, Bosch-Marce M, Nanayakkara A, Savransky V, Fried SK, et al. (2006) Altered metabolic responses to intermittent hypoxia in mice with partial deficiency of hypoxia-inducible factor-1alpha. Physiol Genomics 25: 450-457.

38. Zhang Y, Wang H, Zhang J, Lv J, Huang Y (2013) Positive feedback loop and synergistic effects between hypoxia-inducible factor-2alpha and stearoyl-CoA desaturase-1 promote tumorigenesis in clear cell renal cell carcinoma. Cancer Sci 104: 416-422.

39. Igal RA (2010) Stearoyl-CoA desaturase-1: a novel key player in the mechanisms of cell proliferation, programmed cell death and transformation to cancer. Carcinogenesis 31: 1509-1515.

40. Igal RA (2011) Roles of StearoylCoA Desaturase-1 in the Regulation of Cancer Cell Growth, Survival and Tumorigenesis. Cancers 3: 2462-2477.

41. Gilkes DM, Bajpai S, Chaturvedi P, Wirtz D, Semenza GL (2013) Hypoxia-inducible factor 1 (HIF-1) promotes extracellular matrix remodeling under hypoxic conditions by inducing P4HA1, P4HA2, and PLOD2 expression in fibroblasts. J Biol Chem 288: 10819-10829.

42. Semenza GL, Jiang BH, Leung SW, Passantino R, Concordet JP, et al. (1996) Hypoxia response elements in the aldolase A, enolase 1, and lactate dehydrogenase A gene promoters contain essential binding sites for hypoxia-inducible factor 1. J Biol Chem 271: 32529-32537.

43. Ray R, Miller DM (1991) Cloning and characterization of a human c-myc

promoter-binding protein. Mol Cell Biol 11: 2154-2161.

44. Lo Presti M, Ferro A, Contino F, Mazzarella C, Sbacchi S, et al. (2010) Myc promoter-binding protein-1 (MBP-1) is a novel potential prognostic marker in invasive ductal breast carcinoma. PLoS One 5: e12961.

45. Trojanowicz B, Winkler A, Hammje K, Chen Z, Sekulla C, et al. (2009) Retinoic acid-mediated down-regulation of ENO1/MBP-1 gene products caused decreased invasiveness of the follicular thyroid carcinoma cell lines. J Mol Endocrinol 42: 249-260.

46. Chang YS, Wu W, Walsh G, Hong WK, Mao L (2003) Enolase-alpha is frequently down-regulated in non-small cell lung cancer and predicts aggressive biological behavior. Clin Cancer Res 9: 3641-3644.

47. Ghosh AK, Steele R, Ryerse J, Ray RB (2006) Tumor-suppressive effects of MBP-1 in non-small cell lung cancer cells. Cancer Res 66: 11907-11912.

48. Ejeskar K, Krona C, Caren H, Zaibak F, Li L, et al. (2005) Introduction of in vitro transcribed ENO1 mRNA into neuroblastoma cells induces cell death. BMC Cancer 5: 161.

49. Hsu KW, Hsieh RH, Wu CW, Chi CW, Lee YH, et al. (2009) MBP-1 suppresses growth and metastasis of gastric cancer cells through COX-2. Mol Biol Cell 20: 5127-5137.

50. Gao J, Zhao R, Xue Y, Niu Z, Cui K, et al. (2013) Role of enolase-1 in response to hypoxia in breast cancer: exploring the mechanisms of action. Oncol Rep 29: 1322-1332.

51. Stany MP, Vathipadiekal V, Ozbun L, Stone RL, Mok SC, et al. (2011) Identification of novel therapeutic targets in microdissected clear cell ovarian cancers. PLoS One 6: e21121.

52. Gilkes DM, Chaturvedi P, Bajpai S, Wong CC, Wei H, et al. (2013) Collagen prolyl

hydroxylases are essential for breast cancer metastasis. Cancer Res 73: 3285-3296.

53. Francis P, Namlos HM, Muller C, Eden P, Fernebro J, et al. (2007) Diagnostic and prognostic gene expression signatures in 177 soft tissue sarcomas: hypoxia-induced transcription profile signifies metastatic potential. BMC Genomics 8: 73.

54. Miettinen M (2014) Immunohistochemistry of soft tissue tumours - review with emphasis on 10 markers. Histopathology 64: 101-118.

55. Stella CC, Cazzola M, De Fabritiis P, De Vincentiis A, Gianni AM, et al. (1995) CD34-positive cells: biology and clinical relevance. Haematologica 80: 367-387.

56. Strippoli P, Pelleri MC, Caracausi M, Vitale L, Piovesan A, et al. (2013) An integrated route to identifying new pathogenesis-based therapeutic approaches for trisomy 21 (Down Syndrome) following the thought of Jérôme Lejeune. Science postprint 1: e00010.

57. Siemerink MJ, Klaassen I, Vogels IM, Griffioen AW, Van Noorden CJ, et al. (2012) CD34 marks angiogenic tip cells in human vascular endothelial cell cultures. Angiogenesis 15: 151-163.

58. Mineo TC, Ambrogi V, Baldi A, Rabitti C, Bollero P, et al. (2004) Prognostic impact of VEGF, CD31, CD34, and CD105 expression and tumour vessel invasion after radical surgery for IB-IIA non-small cell lung cancer. J Clin Pathol 57: 591-597.

59. Heimburg S, Oehler MK, Papadopoulos T, Caffier H, Kristen P, et al. (1999) Prognostic relevance of the endothelial marker CD 34 in ovarian cancer. Anticancer Res 19: 2527-2529.

60. Leek RD (2001) The prognostic role of angiogenesis in breast cancer. Anticancer Res 21: 4325-4331.

61. Bettencourt MC, Bauer JJ, Sesterhenn IA, Connelly RR, Moul JW (1998) CD34 immunohistochemical assessment of angiogenesis as a prognostic marker for prostate

cancer recurrence after radical prostatectomy. J Urol 160: 459-465.
62. Thomas X, Archimbaud E, Charrin C, Magaud JP, Fiere D (1995) CD34 expression is associated with major adverse prognostic factors in adult acute lymphoblastic leukemia. Leukemia 9: 249-253.
63. Wen ST, Van Etten RA (1997) The PAG gene product, a stress-induced protein with antioxidant properties, is an Abl SH3-binding protein and a physiological inhibitor of c-Abl tyrosine kinase activity. Genes Dev 11: 2456-2467.
64. Mu ZM, Yin XY, Prochownik EV (2002) Pag, a putative tumor suppressor, interacts with the Myc Box II domain of c-Myc and selectively alters its biological function and target gene expression. J Biol Chem 277: 43175-43184.
65. Riddell JR, Maier P, Sass SN, Moser MT, Foster BA, et al. (2012) Peroxiredoxin 1 stimulates endothelial cell expression of VEGF via TLR4 dependent activation of HIF-1alpha. PLoS One 7: e50394.
66. van Uden P, Kenneth NS, Rocha S (2008) Regulation of hypoxia-inducible factor-1alpha by NF-kappaB. Biochem J 412: 477-484.
67. Hoshino I, Matsubara H, Akutsu Y, Nishimori T, Yoneyama Y, et al. (2007) Tumor suppressor Prdx1 is a prognostic factor in esophageal squamous cell carcinoma patients. Oncol Rep 18: 867-871.
68. Kim JH, Bogner PN, Baek SH, Ramnath N, Liang P, et al. (2008) Up-regulation of peroxiredoxin 1 in lung cancer and its implication as a prognostic and therapeutic target. Clin Cancer Res 14: 2326-2333.
69. Chung KH, Lee DH, Kim Y, Kim TH, Huh JH, et al. (2010) Proteomic identification of overexpressed PRDX 1 and its clinical implications in ovarian carcinoma. J Proteome Res 9: 451-457.
70. Yonglitthipagon P, Pairojkul C, Chamgramol Y, Loukas A, Mulvenna J, et al. (2012)

Prognostic significance of peroxiredoxin 1 and ezrin-radixin-moesin-binding phosphoprotein 50 in cholangiocarcinoma. Hum Pathol 43: 1719-1730.

71. Li J, Yang ZL, Ren X, Zou Q, Yuan Y, et al. (2013) ILK and PRDX1 are prognostic markers in squamous cell/adenosquamous carcinomas and adenocarcinoma of gallbladder. Tumour Biol 34: 359-368.

72. Lee MJ, Kim JY, Suk K, Park JH (2004) Identification of the hypoxia-inducible factor 1 alpha-responsive HGTD-P gene as a mediator in the mitochondrial apoptotic pathway. Mol Cell Biol 24: 3918-3927.

73. Kim JY, Kim SM, Ko JH, Yim JH, Park JH (2006) Interaction of pro-apoptotic protein HGTD-P with heat shock protein 90 is required for induction of mitochondrial apoptotic cascades. FEBS Lett 580: 3270-3275.

74. Lu X, Borchers AG, Jolicoeur C, Rayburn H, Baker JC, et al. (2004) PTK7/CCK-4 is a novel regulator of planar cell polarity in vertebrates. Nature 430: 93-98.

75. Peradziryi H, Kaplan NA, Podleschny M, Liu X, Wehner P, et al. (2011) PTK7/Otk interacts with Wnts and inhibits canonical Wnt signalling. EMBO J 30: 3729-3740.

76. Hayes M, Naito M, Daulat A, Angers S, Ciruna B (2013) Ptk7 promotes non-canonical Wnt/PCP-mediated morphogenesis and inhibits Wnt/beta-catenin-dependent cell fate decisions during vertebrate development. Development 140: 1807-1818.

77. Meng L, Sefah K, O'Donoghue MB, Zhu G, Shangguan D, et al. (2010) Silencing of PTK7 in colon cancer cells: caspase-10-dependent apoptosis via mitochondrial pathway. PLoS One 5: e14018.

78. Gobble RM, Qin LX, Brill ER, Angeles CV, Ugras S, et al. (2011) Expression profiling of liposarcoma yields a multigene predictor of patient outcome and identifies genes that contribute to liposarcomagenesis. Cancer Res 71: 2697-2705.

79. Shin WS, Kwon J, Lee HW, Kang MC, Na HW, et al. (2013) Oncogenic role of protein

tyrosine kinase 7 in esophageal squamous cell carcinoma. Cancer Sci 104: 1120-1126.

80. Lin Y, Zhang LH, Wang XH, Xing XF, Cheng XJ, et al. (2012) PTK7 as a novel marker for favorable gastric cancer patient survival. J Surg Oncol 106: 880-886.

81. Endoh H, Tomida S, Yatabe Y, Konishi H, Osada H, et al. (2004) Prognostic model of pulmonary adenocarcinoma by expression profiling of eight genes as determined by quantitative real-time reverse transcriptase polymerase chain reaction. J Clin Oncol 22: 811-819.

82. Prebet T, Lhoumeau AC, Arnoulet C, Aulas A, Marchetto S, et al. (2010) The cell polarity PTK7 receptor acts as a modulator of the chemotherapeutic response in acute myeloid leukemia and impairs clinical outcome. Blood 116: 2315-2323.

83. Ataseven B, Angerer R, Kates R, Gunesch A, Knyazev P, et al. (2013) PTK7 expression in triple-negative breast cancer. Anticancer Res 33: 3759-3763.

84. Gartner S, Gunesch A, Knyazeva T, Wolf P, Hogel B, et al. (2014) PTK 7 Is a Transforming Gene and Prognostic Marker for Breast Cancer and Nodal Metastasis Involvement. PLoS One 9: e84472.

85. Bae I, Rih JK, Kim HJ, Kang HJ, Haddad B, et al. (2005) BRCA1 regulates gene expression for orderly mitotic progression. Cell Cycle 4: 1641-1666.

86. Pei L, Melmed S (1997) Isolation and characterization of a pituitary tumor-transforming gene (PTTG). Mol Endocrinol 11: 433-441.

87. Bernal JA, Luna R, Espina A, Lazaro I, Ramos-Morales F, et al. (2002) Human securin interacts with p53 and modulates p53-mediated transcriptional activity and apoptosis. Nat Genet 32: 306-311.

88. Minematsu T, Egashira N, Kajiya H, Takei M, Takekoshi S, et al. (2007) PTTG is a secretory protein in human pituitary adenomas and in mouse pituitary tumor cell lines. Endocr Pathol 18: 8-15.

89. Shibata Y, Haruki N, Kuwabara Y, Nishiwaki T, Kato J, et al. (2002) Expression of PTTG (pituitary tumor transforming gene) in esophageal cancer. Jpn J Clin Oncol 32: 233-237.

90. Boelaert K, McCabe CJ, Tannahill LA, Gittoes NJ, Holder RL, et al. (2003) Pituitary tumor transforming gene and fibroblast growth factor-2 expression: potential prognostic indicators in differentiated thyroid cancer. J Clin Endocrinol Metab 88: 2341-2347.

91. Solbach C, Roller M, Fellbaum C, Nicoletti M, Kaufmann M (2004) PTTG mRNA expression in primary breast cancer: a prognostic marker for lymph node invasion and tumor recurrence. Breast 13: 80-81.

92. Zhu X, Mao Z, Na Y, Guo Y, Wang X, et al. (2006) Significance of pituitary tumor transforming gene 1 (PTTG1) in prostate cancer. Anticancer Res 26: 1253-1259.

93. Fujii T, Nomoto S, Koshikawa K, Yatabe Y, Teshigawara O, et al. (2006) Overexpression of pituitary tumor transforming gene 1 in HCC is associated with angiogenesis and poor prognosis. Hepatology 43: 1267-1275.

94. Genkai N, Homma J, Sano M, Tanaka R, Yamanaka R (2006) Increased expression of pituitary tumor-transforming gene (PTTG)-1 is correlated with poor prognosis in glioma patients. Oncol Rep 15: 1569-1574.

95. Winnepenninckx V, Debiec-Rychter M, Belien JA, Fiten P, Michiels S, et al. (2006) Expression and possible role of hPTTG1/securin in cutaneous malignant melanoma. Mod Pathol 19: 1170-1180.

96. Solbach C, Roller M, Eckerdt F, Peters S, Knecht R (2006) Pituitary tumor-transforming gene expression is a prognostic marker for tumor recurrence in squamous cell carcinoma of the head and neck. BMC Cancer 6: 242.

97. Kim JW, Song JY, Lee JM, Lee JK, Lee NW, et al. (2008) Expression of pituitary

tumor-transforming gene in endometrial cancer as a prognostic marker. Int J Gynecol Cancer 18: 1352-1359.

98. Li H, Yin C, Zhang B, Sun Y, Shi L, et al. (2013) PTTG1 promotes migration and invasion of human non-small cell lung cancer cells and is modulated by miR-186. Carcinogenesis 34: 2145-2155.

99. Zhong X, Liu L, Zhao A, Pfeifer GP, Xu X (2005) The abnormal spindle-like, microcephaly-associated (ASPM) gene encodes a centrosomal protein. Cell Cycle 4: 1227-1229.

100. Kato TA, Okayasu R, Jeggo PA, Fujimori A (2011) ASPM influences DNA double-strand break repair and represents a potential target for radiotherapy. Int J Radiat Biol 87: 1189-1195.

101. Lin SY, Pan HW, Liu SH, Jeng YM, Hu FC, et al. (2008) ASPM is a novel marker for vascular invasion, early recurrence, and poor prognosis of hepatocellular carcinoma. Clin Cancer Res 14: 4814-4820.

102. Kabbarah O, Nogueira C, Feng B, Nazarian RM, Bosenberg M, et al. (2010) Integrative genome comparison of primary and metastatic melanomas. PLoS One 5: e10770.

103. Wang WY, Hsu CC, Wang TY, Li CR, Hou YC, et al. (2013) A gene expression signature of epithelial tubulogenesis and a role for ASPM in pancreatic tumor progression. Gastroenterology 145: 1110-1120.

104. Komatsu M, Yoshimaru T, Matsuo T, Kiyotani K, Miyoshi Y, et al. (2013) Molecular features of triple negative breast cancer cells by genome-wide gene expression profiling analysis. Int J Oncol 42: 478-506.

105. Visnyei K, Onodera H, Damoiseaux R, Saigusa K, Petrosyan S, et al. (2011) A molecular screening approach to identify and characterize inhibitors of glioblastoma stem cells. Mol Cancer Ther 10: 1818-1828.

106. Bruning-Richardson A, Bond J, Alsiary R, Richardson J, Cairns DA, et al. (2011) ASPM and microcephalin expression in epithelial ovarian cancer correlates with tumour grade and survival. Br J Cancer 104: 1602-1610.

107. Weinstein J, Jacobsen FW, Hsu-Chen J, Wu T, Baum LG (1994) A novel mammalian protein, p55CDC, present in dividing cells is associated with protein kinase activity and has homology to the Saccharomyces cerevisiae cell division cycle proteins Cdc20 and Cdc4. Mol Cell Biol 14: 3350-3363.

108. Weinstein J (1997) Cell cycle-regulated expression, phosphorylation, and degradation of p55Cdc. A mammalian homolog of CDC20/Fizzy/slp1. J Biol Chem 272: 28501-28511.

109. Kidokoro T, Tanikawa C, Furukawa Y, Katagiri T, Nakamura Y, et al. (2008) CDC20, a potential cancer therapeutic target, is negatively regulated by p53. Oncogene 27: 1562-1571.

110. Ouellet V, Guyot MC, Le Page C, Filali-Mouhim A, Lussier C, et al. (2006) Tissue array analysis of expression microarray candidates identifies markers associated with tumor grade and outcome in serous epithelial ovarian cancer. Int J Cancer 119: 599-607.

111. Kato T, Daigo Y, Aragaki M, Ishikawa K, Sato M, et al. (2012) Overexpression of CDC20 predicts poor prognosis in primary non-small cell lung cancer patients. J Surg Oncol 106: 423-430.

112. Wu WJ, Hu KS, Wang DS, Zeng ZL, Zhang DS, et al. (2013) CDC20 overexpression predicts a poor prognosis for patients with colorectal cancer. J Transl Med 11: 142.

113. Moura IM, Delgado ML, Silva PM, Lopes CA, do Amaral JB, et al. (2013) High CDC20 expression is associated with poor prognosis in oral squamous cell carcinoma. J Oral Pathol Med.

114. Espinosa AM, Alfaro A, Roman-Basaure E, Guardado-Estrada M, Palma I, et al. (2013)

Mitosis is a source of potential markers for screening and survival and therapeutic targets in cervical cancer. PLoS One 8: e55975.

115. Choi JW, Kim Y, Lee JH, Kim YS (2013) High expression of spindle assembly checkpoint proteins CDC20 and MAD2 is associated with poor prognosis in urothelial bladder cancer. Virchows Arch 463: 681-687.

116. Chang DZ, Ma Y, Ji B, Liu Y, Hwu P, et al. (2012) Increased CDC20 expression is associated with pancreatic ductal adenocarcinoma differentiation and progression. J Hematol Oncol 5: 15.

117. Suzuki N, Hazama S, Ueno T, Matsui H, Shindo Y, et al. (2014) A Phase I Clinical Trial of Vaccination With KIF20A-derived Peptide in Combination With Gemcitabine For Patients With Advanced Pancreatic Cancer. J Immunother 37: 36-42.

118. Lee SH, McCormick F, Saya H (2010) Mad2 inhibits the mitotic kinesin MKlp2. J Cell Biol 191: 1069-1077.

119. Mondal G, Baral RN, Roychoudhury S (2006) A new Mad2-interacting domain of Cdc20 is critical for the function of Mad2-Cdc20 complex in the spindle assembly checkpoint. Biochem J 396: 243-253.

120. Groth-Pedersen L, Aits S, Corcelle-Termeau E, Petersen NH, Nylandsted J, et al. (2012) Identification of cytoskeleton-associated proteins essential for lysosomal stability and survival of human cancer cells. PLoS One 7: e45381.

121. Yan GR, Zou FY, Dang BL, Zhang Y, Yu G, et al. (2012) Genistein-induced mitotic arrest of gastric cancer cells by downregulating KIF20A, a proteomics study. Proteomics 12: 2391-2399.

122. Yamashita J, Fukushima S, Jinnin M, Honda N, Makino K, et al. (2012) Kinesin family member 20A is a novel melanoma-associated antigen. Acta Derm Venereol 92: 593-597.

123. Taniuchi K, Nakagawa H, Nakamura T, Eguchi H, Ohigashi H, et al. (2005) Down-regulation of RAB6KIFL/KIF20A, a kinesin involved with membrane trafficking of discs large homologue 5, can attenuate growth of pancreatic cancer cell. Cancer Res 65: 105-112.

124. Garcia CK, Goldstein JL, Pathak RK, Anderson RG, Brown MS (1994) Molecular characterization of a membrane transporter for lactate, pyruvate, and other monocarboxylates: implications for the Cori cycle. Cell 76: 865-873.

125. Kasiappan R, Shih HJ, Chu KL, Chen WT, Liu HP, et al. (2009) Loss of p53 and MCT-1 overexpression synergistically promote chromosome instability and tumorigenicity. Mol Cancer Res 7: 536-548.

126. De Saedeleer CJ, Copetti T, Porporato PE, Verrax J, Feron O, et al. (2012) Lactate activates HIF-1 in oxidative but not in Warburg-phenotype human tumor cells. PLoS One 7: e46571.

127. Sonveaux P, Copetti T, De Saedeleer CJ, Vegran F, Verrax J, et al. (2012) Targeting the lactate transporter MCT1 in endothelial cells inhibits lactate-induced HIF-1 activation and tumor angiogenesis. PLoS One 7: e33418.

128. Zhao Z, Wu MS, Zou C, Tang Q, Lu J, et al. (2014) Downregulation of MCT1 inhibits tumor growth, metastasis and enhances chemotherapeutic efficacy in osteosarcoma through regulation of the NF-kappaB pathway. Cancer Lett 342: 150-158.

129. Miranda-Goncalves V, Honavar M, Pinheiro C, Martinho O, Pires MM, et al. (2013) Monocarboxylate transporters (MCTs) in gliomas: expression and exploitation as therapeutic targets. Neuro Oncol 15: 172-188.

130. Polanski R, Hodgkinson C, Fusi A, Nonaka D, Priest L, et al. (2013) Activity of the Monocarboxylate Transporter 1 inhibitor AZD3965 in Small Cell Lung Cancer. Clin Cancer Res.

131. Curry JM, Tuluc M, Whitaker-Menezes D, Ames JA, Anantharaman A, et al. (2013) Cancer metabolism, stemness and tumor recurrence: MCT1 and MCT4 are functional biomarkers of metabolic symbiosis in head and neck cancer. Cell Cycle 12: 1371-1384.

132. Izumi H, Takahashi M, Uramoto H, Nakayama Y, Oyama T, et al. (2011) Monocarboxylate transporters 1 and 4 are involved in the invasion activity of human lung cancer cells. Cancer Sci 102: 1007-1013.

133. Pinheiro C, Albergaria A, Paredes J, Sousa B, Dufloth R, et al. (2010) Monocarboxylate transporter 1 is up-regulated in basal-like breast carcinoma. Histopathology 56: 860-867.

134. Pinheiro C, Longatto-Filho A, Scapulatempo C, Ferreira L, Martins S, et al. (2008) Increased expression of monocarboxylate transporters 1, 2, and 4 in colorectal carcinomas. Virchows Arch 452: 139-146.

135. Shimoyama Y, Akihara Y, Kirat D, Iwano H, Hirayama K, et al. (2007) Expression of monocarboxylate transporter 1 in oral and ocular canine melanocytic tumors. Vet Pathol 44: 449-457.

136. Masuda M, Maruyama T, Ohta T, Ito A, Hayashi T, et al. (2010) CADM1 interacts with Tiam1 and promotes invasive phenotype of human T-cell leukemia virus type I-transformed cells and adult T-cell leukemia cells. J Biol Chem 285: 15511-15522.

137. Masuda M, Yageta M, Fukuhara H, Kuramochi M, Maruyama T, et al. (2002) The tumor suppressor protein TSLC1 is involved in cell-cell adhesion. J Biol Chem 277: 31014-31019.

138. Lu B, Di W, Wang H, Ma H, Li J, et al. (2012) Tumor suppressor TSLC1 is implicated in cell proliferation, invasion and apoptosis in laryngeal squamous cell carcinoma by regulating Akt signaling pathway. Tumour Biol 33: 2007-2017.

139. Fukami T, Fukuhara H, Kuramochi M, Maruyama T, Isogai K, et al. (2003) Promoter methylation of the TSLC1 gene in advanced lung tumors and various cancer cell lines. Int J Cancer 107: 53-59.

140. He G, Lei W, Wang S, Xiao R, Guo K, et al. (2012) Overexpression of tumor suppressor TSLC1 by a survivin-regulated oncolytic adenovirus significantly inhibits hepatocellular carcinoma growth. J Cancer Res Clin Oncol 138: 657-670.

141. Qin L, Zhu W, Xu T, Hao Y, Zhang Z, et al. (2007) Effect of TSLC1 gene on proliferation, invasion and apoptosis of human hepatocellular carcinoma cell line HepG2. J Huazhong Univ Sci Technolog Med Sci 27: 535-537.

142. Houshmandi SS, Surace EI, Zhang HB, Fuller GN, Gutmann DH (2006) Tumor suppressor in lung cancer-1 (TSLC1) functions as a glioma tumor suppressor. Neurology 67: 1863-1866.

143. Uchino K, Ito A, Wakayama T, Koma Y, Okada T, et al. (2003) Clinical implication and prognostic significance of the tumor suppressor TSLC1 gene detected in adenocarcinoma of the lung. Cancer 98: 1002-1007.

144. Malik TH, Shoichet SA, Latham P, Kroll TG, Peters LL, et al. (2001) Transcriptional repression and developmental functions of the atypical vertebrate GATA protein TRPS1. EMBO J 20: 1715-1725.

145. Chen JQ, Litton J, Xiao L, Zhang HZ, Warneke CL, et al. (2010) Quantitative immunohistochemical analysis and prognostic significance of TRPS-1, a new GATA transcription factor family member, in breast cancer. Horm Cancer 1: 21-33.

146. Bao Y, Ruan LJ, Mo JF (2013) Low trichorhinophalangeal syndrome 1 gene transcript levels in basal-like breast cancer associate with mesenchymal-to-epithelial transition. Chin Med Sci J 28: 129-134.

147. Hong J, Sun J, Huang T (2013) Increased expression of TRPS1 affects tumor

progression and correlates with patients' prognosis of colon cancer. Biomed Res Int 2013: 454085.

148. Sipley JD, Menninger JC, Hartley KO, Ward DC, Jackson SP, et al. (1995) Gene for the catalytic subunit of the human DNA-activated protein kinase maps to the site of the XRCC7 gene on chromosome 8. Proc Natl Acad Sci USA 92: 7515-7519.

149. Tomimatsu N, Mukherjee B, Burma S (2009) Distinct roles of ATR and DNA-PKcs in triggering DNA damage responses in ATM-deficient cells. EMBO Rep 10: 629-635.

150. Hill R, Madureira PA, Waisman DM, Lee PW (2011) DNA-PKCS binding to p53 on the p21WAF1/CIP1 promoter blocks transcription resulting in cell death. Oncotarget 2: 1094-1108.

151. Someya M, Sakata KI, Matsumoto Y, Kamdar RP, Kai M, et al. (2011) The association of DNA-dependent protein kinase activity of peripheral blood lymphocytes with prognosis of cancer. Br J Cancer 104: 1724-1729.

152. Xing J, Wu X, Vaporciyan AA, Spitz MR, Gu J (2008) Prognostic significance of ataxia-telangiectasia mutated, DNA-dependent protein kinase catalytic subunit, and Ku heterodimeric regulatory complex 86-kD subunit expression in patients with nonsmall cell lung cancer. Cancer 112: 2756-2764.

153. Willmore E, Elliott SL, Mainou-Fowler T, Summerfield GP, Jackson GH, et al. (2008) DNA-dependent protein kinase is a therapeutic target and an indicator of poor prognosis in B-cell chronic lymphocytic leukemia. Clin Cancer Res 14: 3984-3992.

154. Malumbres M, Barbacid M (2005) Mammalian cyclin-dependent kinases. Trends Biochem Sci 30: 630-641.

155. Bunz F, Dutriaux A, Lengauer C, Waldman T, Zhou S, et al. (1998) Requirement for p53 and p21 to sustain G2 arrest after DNA damage. Science 282: 1497-1501.

156. Wolowiec D, Berger F, Ffrench P, Bryon PA, Ffrench M (1999) CDK1 and cyclin A

expression is linked to cell proliferation and associated with prognosis in non-Hodgkin's lymphomas. Leuk Lymphoma 35: 147-157.

157. Perumal D, Singh S, Yoder SJ, Bloom GC, Chellappan SP (2012) A novel five gene signature derived from stem-like side population cells predicts overall and recurrence-free survival in NSCLC. PLoS One 7: e43589.

158. Willder JM, Heng SJ, McCall P, Adams CE, Tannahill C, et al. (2013) Androgen receptor phosphorylation at serine 515 by Cdk1 predicts biochemical relapse in prostate cancer patients. Br J Cancer 108: 139-148.

159. Linton A, Cheng YY, Griggs K, Kirschner MB, Gattani S, et al. (2013) An RNAi-based screen reveals PLK1, CDK1 and NDC80 as potential therapeutic targets in malignant pleural mesothelioma. Br J Cancer.

160. Kim SJ, Nakayama S, Miyoshi Y, Taguchi T, Tamaki Y, et al. (2008) Determination of the specific activity of CDK1 and CDK2 as a novel prognostic indicator for early breast cancer. Ann Oncol 19: 68-72.

161. Jeng JC, Lin YM, Lin CH, Shih HM (2009) Cdh1 controls the stability of TACC3. Cell Cycle 8: 3529-3536.

162. Partch CL, Gardner KH (2011) Coactivators necessary for transcriptional output of the hypoxia inducible factor, HIF, are directly recruited by ARNT PAS-B. Proc Natl Acad Sci USA 108: 7739-7744.

163. Piekorz RP, Hoffmeyer A, Duntsch CD, McKay C, Nakajima H, et al. (2002) The centrosomal protein TACC3 is essential for hematopoietic stem cell function and genetically interfaces with p53-regulated apoptosis. EMBO J 21: 653-664.

164. Jung CK, Jung JH, Park GS, Lee A, Kang CS, et al. (2006) Expression of transforming acidic coiled-coil containing protein 3 is a novel independent prognostic marker in non-small cell lung cancer. Pathol Int 56: 503-509.

165. Yim EK, Tong SY, Ho EM, Bae JH, Um SJ, et al. (2009) Anticancer effects on TACC3 by treatment of paclitaxel in HPV-18 positive cervical carcinoma cells. Oncol Rep 21: 549-557.

166. Duncan CG, Killela PJ, Payne CA, Lampson B, Chen WC, et al. (2010) Integrated genomic analyses identify ERRFI1 and TACC3 as glioblastoma-targeted genes. Oncotarget 1: 265-277.

167. Shao GZ, Zhou RL, Zhang QY, Zhang Y, Liu JJ, et al. (2003) Molecular cloning and characterization of LAPTM4B, a novel gene upregulated in hepatocellular carcinoma. Oncogene 22: 5060-5069.

168. Li Y, Zhang Q, Tian R, Wang Q, Zhao JJ, et al. (2011) Lysosomal transmembrane protein LAPTM4B promotes autophagy and tolerance to metabolic stress in cancer cells. Cancer Res 71: 7481-7489.

169. Li L, Wei XH, Pan YP, Li HC, Yang H, et al. (2010) LAPTM4B: a novel cancer-associated gene motivates multidrug resistance through efflux and activating PI3K/AKT signaling. Oncogene 29: 5785-5795.

170. Yang H, Xiong F, Wei X, Yang Y, McNutt MA, et al. (2010) Overexpression of LAPTM4B-35 promotes growth and metastasis of hepatocellular carcinoma in vitro and in vivo. Cancer Lett 294: 236-244.

171. Xiao M, Jia S, Wang H, Wang J, Huang Y, et al. (2013) Overexpression of LAPTM4B: an independent prognostic marker in breast cancer. J Cancer Res Clin Oncol 139: 661-667.

172. Kang Y, Yin M, Jiang W, Zhang H, Xia B, et al. (2012) Overexpression of LAPTM4B-35 is associated with poor prognosis in colorectal carcinoma. Am J Surg 204: 677-683.

173. Yin M, Lou C, Zhang W, Meng F, Zhang H, et al. (2012) LAPTM4B overexpression is a

novel independent prognostic marker for metastatic ovarian tumors. Int J Gynecol Cancer 22: 54-62.

174. Zhou L, He XD, Cui QC, Zhou WX, Qu Q, et al. (2008) Expression of LAPTM4B-35: a novel marker of progression, invasiveness and poor prognosis of extrahepatic cholangiocarcinoma. Cancer Lett 264: 209-217.

175. Krishnamachary B, Berg-Dixon S, Kelly B, Agani F, Feldser D, et al. (2003) Regulation of colon carcinoma cell invasion by hypoxia-inducible factor 1. Cancer Res 63: 1138-1143.

176. Lou X, Han X, Jin C, Tian W, Yu W, et al. (2013) SOX2 targets fibronectin 1 to promote cell migration and invasion in ovarian cancer: new molecular leads for therapeutic intervention. OMICS 17: 510-518.

177. Helleman J, Jansen MP, Ruigrok-Ritstier K, van Staveren IL, Look MP, et al. (2008) Association of an extracellular matrix gene cluster with breast cancer prognosis and endocrine therapy response. Clin Cancer Res 14: 5555-5564.

178. Waalkes S, Atschekzei F, Kramer MW, Hennenlotter J, Vetter G, et al. (2010) Fibronectin 1 mRNA expression correlates with advanced disease in renal cancer. BMC Cancer 10: 503.

179. Nusinow DA, Sharp JA, Morris A, Salas S, Plath K, et al. (2007) The histone domain of macroH2A1 contains several dispersed elements that are each sufficient to direct enrichment on the inactive X chromosome. J Mol Biol 371: 11-18.

180. Riballo E, Kuhne M, Rief N, Doherty A, Smith GC, et al. (2004) A pathway of double-strand break rejoining dependent upon ATM, Artemis, and proteins locating to gamma-H2AX foci. Mol Cell 16: 715-724.

181. Blattmann C, Oertel S, Thiemann M, Weber KJ, Schmezer P, et al. (2012) Suberoylanilide hydroxamic acid affects gammaH2AX expression in osteosarcoma,

atypical teratoid rhabdoid tumor and normal tissue cell lines after irradiation. Strahlenther Onkol 188: 168-176.
182. Li X, Kuang J, Shen Y, Majer MM, Nelson CC, et al. (2012) The atypical histone macroH2A1.2 interacts with HER-2 protein in cancer cells. J Biol Chem 287: 23171-23183.
183. Sengupta S, Robles AI, Linke SP, Sinogeeva NI, Zhang R, et al. (2004) Functional interaction between BLM helicase and 53BP1 in a Chk1-mediated pathway during S-phase arrest. J Cell Biol 166: 801-813.
184. Fernandez-Capetillo O, Chen HT, Celeste A, Ward I, Romanienko PJ, et al. (2002) DNA damage-induced G2-M checkpoint activation by histone H2AX and 53BP1. Nat Cell Biol 4: 993-997.
185. Ward IM, Minn K, Jorda KG, Chen J (2003) Accumulation of checkpoint protein 53BP1 at DNA breaks involves its binding to phosphorylated histone H2AX. J Biol Chem 278: 19579-19582.
186. Mallery DL, Vandenberg CJ, Hiom K (2002) Activation of the E3 ligase function of the BRCA1/BARD1 complex by polyubiquitin chains. EMBO J 21: 6755-6762.
187. Paull TT, Rogakou EP, Yamazaki V, Kirchgessner CU, Gellert M, et al. (2000) A critical role for histone H2AX in recruitment of repair factors to nuclear foci after DNA damage. Curr Biol 10: 886-895.
188. Chen A, Kleiman FE, Manley JL, Ouchi T, Pan ZQ (2002) Autoubiquitination of the BRCA1*BARD1 RING ubiquitin ligase. J Biol Chem 277: 22085-22092.
189. Fragkos M, Jurvansuu J, Beard P (2009) H2AX is required for cell cycle arrest via the p53/p21 pathway. Mol Cell Biol 29: 2828-2840.
190. Sporn JC, Jung B (2012) Differential regulation and predictive potential of MacroH2A1 isoforms in colon cancer. Am J Pathol 180: 2516-2526.

191. Matthaios D, Foukas PG, Kefala M, Hountis P, Trypsianis G, et al. (2012) gamma-H2AX expression detected by immunohistochemistry correlates with prognosis in early operable non-small cell lung cancer. Onco Targets Ther 5: 309-314.
192. Nagelkerke A, van Kuijk SJ, Sweep FC, Nagtegaal ID, Hoogerbrugge N, et al. (2011) Constitutive expression of gamma-H2AX has prognostic relevance in triple negative breast cancer. Radiother Oncol 101: 39-45.
193. Brunner AH, Hinterholzer S, Riss P, Heinze G, Weiss K, et al. (2011) Expression of gamma-H2AX in endometrial carcinomas: an immunohistochemical study with p53. Gynecol Oncol 121: 206-211.
194. Schindler C, Fu XY, Improta T, Aebersold R, Darnell JE, Jr. (1992) Proteins of transcription factor ISGF-3: one gene encodes the 91-and 84-kDa ISGF-3 proteins that are activated by interferon alpha. Proc Natl Acad Sci USA 89: 7836-7839.
195. Darnell JE, Jr., Kerr IM, Stark GR (1994) Jak-STAT pathways and transcriptional activation in response to IFNs and other extracellular signaling proteins. Science 264: 1415-1421.
196. Ihle JN, Kerr IM (1995) Jaks and Stats in signaling by the cytokine receptor superfamily. Trends Genet 11: 69-74.
197. Stark GR, Kerr IM, Williams BR, Silverman RH, Schreiber RD (1998) How cells respond to interferons. Annu Rev Biochem 67: 227-264.
198. Adamkova L, Souckova K, Kovarik J (2007) Transcription protein STAT1: biology and relation to cancer. Folia Biol (Praha) 53: 1-6.
199. Kramer OH, Baus D, Knauer SK, Stein S, Jager E, et al. (2006) Acetylation of Stat1 modulates NF-kappaB activity. Genes Dev 20: 473-485.
200. Townsend PA, Scarabelli TM, Davidson SM, Knight RA, Latchman DS, et al. (2004) STAT-1 interacts with p53 to enhance DNA damage-induced apoptosis. J Biol Chem

279: 5811-5820.

201. Ivanov SV, Salnikow K, Ivanova AV, Bai L, Lerman MI (2007) Hypoxic repression of STAT1 and its downstream genes by a pVHL/HIF-1 target DEC1/STRA13. Oncogene 26: 802-812.

202. Widschwendter A, Tonko-Geymayer S, Welte T, Daxenbichler G, Marth C, et al. (2002) Prognostic significance of signal transducer and activator of transcription 1 activation in breast cancer. Clin Cancer Res 8: 3065-3074.

203. Watanabe G, Kaganoi J, Imamura M, Shimada Y, Itami A, et al. (2001) Progression of esophageal carcinoma by loss of EGF-STAT1 pathway. Cancer J 7: 132-139.

204. Chen HY, Yu SL, Chen CH, Chang GC, Chen CY, et al. (2007) A five-gene signature and clinical outcome in non-small-cell lung cancer. N Engl J Med 356: 11-20.

205. Gordziel C, Bratsch J, Moriggl R, Knosel T, Friedrich K (2013) Both STAT1 and STAT3 are favourable prognostic determinants in colorectal carcinoma. Br J Cancer 109: 138-146.

206. Hix LM, Karavitis J, Khan MW, Shi YH, Khazaie K, et al. (2013) Tumor STAT1 transcription factor activity enhances breast tumor growth and immune suppression mediated by myeloid-derived suppressor cells. J Biol Chem 288: 11676-11688.

207. Dong J, Li J, Liu SM, Feng XY, Chen S, et al. (2013) CD33(+)/p-STAT1(+) double-positive cell as a prognostic factor for stage IIIa gastric cancer. Med Oncol 30: 442.

208. Couet J, Sargiacomo M, Lisanti MP (1997) Interaction of a receptor tyrosine kinase, EGF-R, with caveolins. Caveolin binding negatively regulates tyrosine and serine/threonine kinase activities. J Biol Chem 272: 30429-30438.

209. Goetz JG, Lajoie P, Wiseman SM, Nabi IR (2008) Caveolin-1 in tumor progression: the good, the bad and the ugly. Cancer Metastasis Rev 27: 715-735.

210. Lee H, Park DS, Wang XB, Scherer PE, Schwartz PE, et al. (2002) Src-induced phosphorylation of caveolin-2 on tyrosine 19. Phospho-caveolin-2 (Tyr(P)19) is localized near focal adhesions, remains associated with lipid rafts/caveolae, but no longer forms a high molecular mass hetero-oligomer with caveolin-1. J Biol Chem 277: 34556-34567.

211. Treff NR, Pouchnik D, Dement GA, Britt RL, Reeves R (2004) High-mobility group A1a protein regulates Ras/ERK signaling in MCF-7 human breast cancer cells. Oncogene 23: 777-785.

212. Quann K, Gonzales DM, Mercier I, Wang C, Sotgia F, et al. (2013) Caveolin-1 is a negative regulator of tumor growth in glioblastoma and modulates chemosensitivity to temozolomide. Cell Cycle 12: 1510-1520.

213. Ando T, Ishiguro H, Kimura M, Mitsui A, Mori Y, et al. (2007) The overexpression of caveolin-1 and caveolin-2 correlates with a poor prognosis and tumor progression in esophageal squamous cell carcinoma. Oncol Rep 18: 601-609.

214. Sagara Y, Mimori K, Yoshinaga K, Tanaka F, Nishida K, et al. (2004) Clinical significance of Caveolin-1, Caveolin-2 and HER2/neu mRNA expression in human breast cancer. Br J Cancer 91: 959-965.

215. Nimri L, Barak H, Graeve L, Schwartz B (2013) Restoration of caveolin-1 expression suppresses growth, membrane-type-4 metalloproteinase expression and metastasis-associated activities in colon cancer cells. Mol Carcinog 52: 859-870.

216. Parat MO, Riggins GJ (2012) Caveolin-1, caveolae, and glioblastoma. Neuro Oncol 14: 679-688.

217. Pancotti F, Roncuzzi L, Maggiolini M, Gasperi-Campani A (2012) Caveolin-1 silencing arrests the proliferation of metastatic lung cancer cells through the inhibition of STAT3 signaling. Cell Signal 24: 1390-1397.

218. Jones JI, Clemmons DR (1995) Insulin-like growth factors and their binding proteins: biological actions. Endocr Rev 16: 3-34.

219. LeRoith D, Werner H, Beitner-Johnson D, Roberts CT, Jr. (1995) Molecular and cellular aspects of the insulin-like growth factor I receptor. Endocr Rev 16: 143-163.

220. Rechler MM (1993) Insulin-like growth factor binding proteins. Vitam Horm 47: 1-114.

221. Moreno MJ, Ball M, Andrade MF, McDermid A, Stanimirovic DB (2006) Insulin-like growth factor binding protein-4 (IGFBP-4) is a novel anti-angiogenic and anti-tumorigenic mediator secreted by dibutyryl cyclic AMP (dB-cAMP)-differentiated glioblastoma cells. Glia 53: 845-857.

222. Diehl D, Hoeflich A, Wolf E, Lahm H (2004) Insulin-like growth factor (IGF)-binding protein-4 inhibits colony formation of colorectal cancer cells by IGF-independent mechanisms. Cancer Res 64: 1600-1603.

223. Ueno K, Hirata H, Majid S, Tabatabai ZL, Hinoda Y, et al. (2011) IGFBP-4 activates the Wnt/beta-catenin signaling pathway and induces M-CAM expression in human renal cell carcinoma. Int J Cancer 129: 2360-2369.

224. Sato H, Sakaeda M, Ishii J, Kashiwagi K, Shimoyamada H, et al. (2011) Insulin-like growth factor binding protein-4 gene silencing in lung adenocarcinomas. Pathol Int 61: 19-27.
